# Glutamatergic Medications for Obsessive-Compulsive and Related Disorders: A Systematic Review and Meta-Analysis

**DOI:** 10.1001/jamanetworkopen.2024.52963

**Published:** 2025-01-02

**Authors:** David R. A. Coelho, Chen Yang, Armiel Suriaga, Justen Manasa, Paul A. Bain, Willians Fernando Vieira, Stefania Papatheodorou, Joshua D. Salvi

**Affiliations:** 1 Harvard T.H. Chan School of Public Health, Boston, Massachusetts; 2 Countway Library, Harvard Medical School, Boston, Massachusetts; 3 Department of Anatomy, Institute of Biomedical Sciences, University of São Paulo, São Paulo, SP, Brazil; 4 Laboratory of Neuroimmune Interface of Pain Research, Faculdade São Leopoldo Mandic, Instituto São Leopoldo Mandic, Campinas, SP, Brazil; 5 Deparment of Epidemiology, Harvard T.H. Chan School of Public Health, Boston, Massachusetts; 6 Department of Biostatistics and Epidemiology, Rutgers School of Public Health, Piscataway, New Jersey; 7 Center for OCD and Related Disorders, Massachusetts General Hospital, Boston; 8 Department of Psychiatry, Harvard Medical School, Boston, Massachusetts; 9 McLean Hospital, Belmont, Massachusetts

## Abstract

**Importance:**

Obsessive-compulsive and related disorders (OCRDs) encompass various neuropsychiatric conditions that cause significant distress and impair daily functioning. Although standard treatments are often effective, approximately 60% of patients may not respond adequately, underscoring the need for novel therapeutic approaches.

**Objective:**

To evaluate improvement in OCRD symptoms associated with glutamatergic medications as monotherapy or as augmentation to selective serotonin reuptake inhibitors, with a focus on double-blind, placebo-controlled randomized clinical trials (RCTs).

**Data Sources:**

Electronic searches were conducted in PubMed, Embase, PsycINFO, Web of Science, and Cochrane Central Register of Controlled Trials on October 16, 2024, without date limits.

**Study Selection:**

Two investigators independently screened records to identify double-blind RCTs comparing glutamatergic medications with placebo for patients with OCRDs regardless of age, sex, gender, or refractoriness. Abstracts, study protocols, non-English studies, and trials involving augmentation to psychotherapy were excluded.

**Data Extraction and Synthesis:**

Data were extracted and synthesized using random-effects meta-analyses. Subgroup analysis was conducted based on type of OCRD, population, refractoriness of OCRD, augmentation strategy, risk of bias, and type of glutamatergic medication. Sensitivity analysis was performed using a leave-one-out approach.

**Main Outcomes and Measures:**

Improvement in OCRD symptoms was measured by standardized mean difference (Cohen *d*). Improvement in obsessive-compulsive disorder (OCD) symptoms was measured by mean difference (reduction in Yale-Brown Obsessive Compulsive Scale [Y-BOCS] scores).

**Results:**

A total of 27 RCTs (1369 participants; mean [SD] age, 31.5 [7.8] years; 65.6% female) were included. Glutamatergic medications showed a large effect size in improving OCRD symptoms (Cohen *d* = −0.80 [95% CI, −1.13 to −0.47]; low certainty of evidence). In the 23 OCD-specific RCTs, glutamatergic medications demonstrated a significant mean reduction in Y-BOCS scores (mean difference, −4.17 [95% CI, −5.82 to −2.52]; moderate certainty of evidence).

**Conclusions and Relevance:**

These findings indicate that glutamatergic medications may be effective in treating OCRDs, particularly OCD. However, high heterogeneity and potential publication bias necessitate cautious interpretation. Further research with larger sample sizes is needed to explore dose-dependent effects, additional OCRD subtypes, and other promising glutamatergic medications.

## Key Points

**Question:**

Are glutamatergic medications associated with improvement in symptoms of obsessive-compulsive and related disorders (OCRDs)?

**Findings:**

In this systematic review and meta-analysis of 27 randomized clinical trials involving 1369 individuals, glutamatergic medications were associated with significant improvement in symptoms of OCRDs, with a large effect size observed. For obsessive-compulsive disorder (OCD) specifically, these medications were associated with a significant mean reduction in Yale-Brown Obsessive Compulsive Scale scores.

**Meaning:**

This study suggests that glutamatergic medications show promise as effective treatments for OCRDs, particularly OCD.

## Teaser

This systematic review and meta-analysis evaluates the association of glutamatergic medications with symptoms of obsessive-compulsive and related disorders as monotherapy or as augmentation to selective serotonin reuptake inhibitors.

## Introduction

Obsessive-compulsive and related disorders (OCRDs) encompass a spectrum of neuropsychiatric conditions characterized by excessive and persistent obsessions or compulsions.^1^ This group includes obsessive-compulsive disorder (OCD), body dysmorphic disorder (BDD), skin-picking disorder (excoriation disorder), trichotillomania (hair pulling), and hoarding disorder, among others, all of which cause significant distress and impair daily functioning.^1,2,3,4^ Obsessive-compulsive and related disorders affect approximately 2% to 3% of the US population, including both children and adults.^5,6,7^ Standard treatments, including selective serotonin reuptake inhibitors (SSRIs), clomipramine, and cognitive behavioral therapy, are often effective.^8^ However, approximately 60% of patients, particularly those with OCD, may not respond adequately to SSRIs as monotherapy, highlighting the need for novel therapeutic approaches.^9^

Glutamatergic dysfunction, particularly in cortico-striatal-thalamo-cortical circuits, has been increasingly implicated in the pathophysiology of these disorders.^10,11^ Studies have expanded our understanding of OCRDs, revealing dysfunctions in neurobiological circuits involving the orbitofrontal cortex, anterior cingulate cortex, and basal ganglia.^12,13,14,15^ These findings have led to a growing interest in the role of the glutamatergic system in OCRDs.^16,17^ Consequently, glutamatergic medications, with their ability to modulate glutamate neurotransmission and receptor activity, have emerged as promising adjunctive treatments.^18^ Agents such as *N*-acetylcysteine (NAC) and memantine have shown potential for OCD.^19,20^

Previous meta-analyses have focused primarily on specific groups of OCRDs, such as the use of glutamatergic medications solely in OCD^21,22^ or focusing on specific medications such as NAC^23,24^ and memantine.^25^ These studies have not comprehensively assessed the broader spectrum of OCRDs or considered subgroup analyses based on important clinical characteristics. This systematic review and meta-analysis aimed to evaluate the outcomes associated with glutamatergic medications in treating OCRDs by focusing on double-blind, placebo-controlled, randomized clinical trials (RCTs) across a diverse range of OCRDs.

## Methods

This systematic review was prepared following the Preferred Reporting Items for Systematic Reviews and Meta-Analyses (PRISMA) reporting guideline. The protocol was preregistered in PROSPERO (CRD42023472430).

### Data Sources

Electronic searches were conducted with the assistance of a medical librarian (P.A.B.) in PubMed (NCBI), Embase (Elsevier), PsycINFO (EBSCO), Web of Science Core Collection (Clarivate), and the Cochrane Central Register of Controlled Trials (Wiley). The search strategy was designed to identify records representing RCTs of glutamatergic medications for improving symptoms in patients with OCRDs. The searches included controlled vocabulary terms when available and were carried out on October 16, 2024, without date limits. The full search strategy is provided in eTables 1 to 5 in Supplement 1.

### Study Selection

Four investigators (D.R.A.C., C.Y., A.S., and J.M.) independently screened the titles, abstracts, and full texts in pairs to identify studies that met inclusion criteria. Discrepancies were resolved by consensus among all reviewers. Covidence software^26^ was used for the screening and study selection process. Inclusion criteria were (1) studies including populations with OCRDs as defined by the *Diagnostic and Statistical Manual of Mental Disorders* (Fifth Edition) (*DSM-5*)^1^: OCD, BDD, skin-picking disorder (excoriation disorder), trichotillomania (hair-pulling disorder or trichobezoar), hoarding disorder (pathological collecting), substance- or medication-induced OCRD, OCRD due to another medical disorder, other specified OCRD, or unspecified OCRD; (2) studies testing glutamatergic medications, such as agmatine, amantadine, d-cycloserine, dextromethorphan, esketamine, gabapentin, glycine, ketamine, lamotrigine, l-carnosine, memantine, minocycline, modafinil, NAC, pregabalin, riluzole, sarcosine, or topiramate; (3) double-blind RCTs with intervention and placebo groups; and (4) studies assessing glutamatergic medications as monotherapy or as augmentation to an SSRI to improve OCRD symptoms. Exclusion criteria were (1) case series, abstracts, reviews, study protocols, or non–peer-reviewed articles; (2) studies involving glutamatergic medications in augmentation to any type of psychotherapy during the trial; (3) non-English studies; and (4) studies where outcome data could not be extracted from text, tables, or figures.

### Data Extraction

Four investigators (D.R.A.C., C.Y., A.S., and J.M.) independently extracted data in pairs using a standardized spreadsheet. Extracted data included first author, publication year, country, sample size for intervention and control groups, study population, type of OCRD, refractoriness of OCRD, age in mean (SD) values or median and age range when the mean age was not reported, male sex (%), mean (SD) years living with OCRD, comorbid psychiatric disorders, type of glutamatergic medication with dose and duration, augmentation strategy (whether the study randomized the population to an SSRI), type of control, outcome measure, and adverse effects. Disagreements were resolved through consensus discussions.

### Outcomes

Outcomes were improvements in OCRD symptoms, evaluated using total scores including (1) the Yale-Brown Obsessive Compulsive Scale (Y-BOCS)^27^ and its variation, the Children’s Yale-Brown Obsessive Compulsive Scale (CY-BOCS),^28^ for OCD and BDD symptoms; (2) the Massachusetts General Hospital Hairpulling Scale (MGH-HPS)^29^ for trichotillomania symptoms; (3) the Skin Picking Scale^30^ or the Y-BOCS modified for neurotic excoriation (NE-YBOCS)^31^ for skin picking disorders; and (4) the Saving Inventory–Revised^32^ for hoarding symptoms. Given that the Y-BOCS is the criterion standard for OCD, we expected to find more studies using this scale.^27^ The Y-BOCS is a clinician-administered scale that assesses the severity of obsessive-compulsive symptoms, consisting of 10 items—5 for obsessions and 5 for compulsions—with total scores ranging from 0 to 40, where higher scores indicate greater symptom severity.^27^

### Statistical Analysis

In the meta-analysis of glutamatergic medications for OCRD symptoms, effect sizes were determined using standardized mean differences (Cohen *d*) to accommodate the diverse measurement methods used across studies, including the Y-BOCS, CY-BOCS, MGH-HPS, and NE-YBOCS.^33^ Differences in means and SDs from baseline to after treatment were either directly extracted from the studies or calculated according to the Cochrane Handbook.^34^ For the meta-analysis of glutamatergic medications for OCD symptoms (excluding the studies on trichotillomania and skin-picking disorder), mean differences were estimated using mean (SD) values at the study end point, as all included studies were RCTs. The mean difference was determined by comparing the mean scores at the study end point between the intervention and control groups. Each estimate was paired with a 95% CI. All statistical analyses were conducted using a random-effects model with the restricted maximum likelihood (REML) method to account for heterogeneity across the included studies.^34^ The sample size for each study arm was extracted based on the initial sample size at randomization (intention-to-treat analysis) to maintain the balanced consideration of potential confounders inherent in RCTs.

Sensitivity analysis was performed using a leave-one-out analysis. Subgroup analyses for the improvement in OCRD symptoms were conducted based on several criteria to assess potential effect modifications: type of OCRD (OCD, skin picking, or trichotillomania); population (children and adolescents or adults); refractoriness of OCRD (yes or no); augmentation strategy (tested as monotherapy or tested as augmentation to an SSRI); risk of bias (low risk, some concerns, or high risk); and type of glutamatergic medication for medications with at least 2 studies (NAC, memantine, lamotrigine, topiramate, or riluzole). Separate subgroup analyses were conducted specifically for OCD (population, refractoriness of OCD, augmentation strategy, risk of bias, and type of glutamatergic medication for medications with at least 2 studies). Univariate meta-regression analysis was also conducted for continuous variables (mean age of population, mean years living with OCRD or OCD, or weeks of treatment). Heterogeneity was evaluated using the *Q* statistic and *I*^2^ statistics.^34^ Publication bias was assessed with funnel plots and the Egger test.^35^ Statistical analyses were performed using Stata, version 18.0 (StataCorp LLC), with *P* < .05 from 2-sided tests indicating statistical significance.

Risk of bias was independently assessed by 4 investigators (D.R.A.C., C.Y., A.S., and J.M.) using the Cochrane Risk of Bias tool for RCTs (RoB2).^34^ Studies were categorized as having a low risk of bias, some concerns, or a high risk of bias based on 5 domains: randomization process, deviations from the intended intervention, missing outcome data, measurement of the outcome, and selection of reported results. In addition, the certainty of evidence was evaluated by 3 investigators (D.R.A.C., C.Y., and A.S.) using the Grading of Recommendations, Assessment, Development, and Evaluations (GRADE) framework.^34^ The level of certainty for each outcome was categorized as high, moderate, low, or very low, based on 5 components: risk of bias, inconsistency, indirectness, imprecision, and publication bias. All differences among the investigators were resolved by consensus.

## Results

### Study Selection and Characteristics

**Figure 1.**
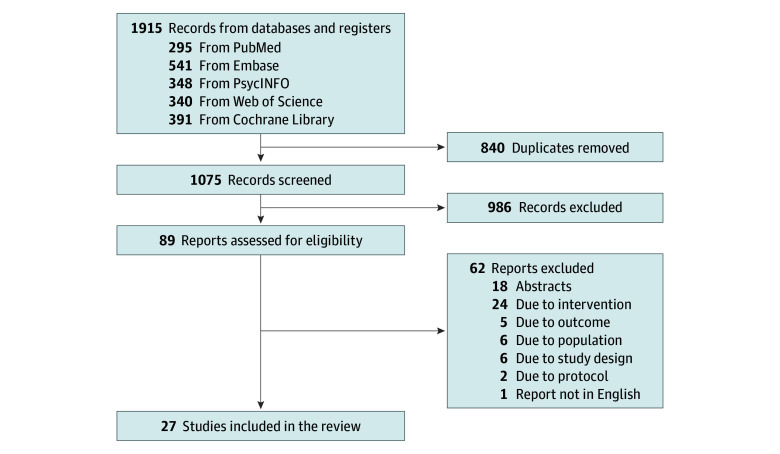
Study Flow Diagram

From the initial 1915 records retrieved, 840 duplicates were removed. Subsequently, 1075 records were screened by title and abstract, resulting in the exclusion of 986 records that did not meet the inclusion criteria. A total of 89 reports were retrieved and examined in full text, of which 27 reports representing 27 independent studies were included in the review (23 studies for OCD).^36,37,38,39,40,41,42,43,44,45,46,47,48,49,50,51,52,53,54,55,56,57,58,59,60,61,62^ The study selection process is illustrated in Figure 1, and all excluded reports with reasons are detailed in eTable 6 in Supplement 1.

Across the 27 studies included,^36,37,38,39,40,41,42,43,44,45,46,47,48,49,50,51,52,53,54,55,56,57,58,59,60,61,62^ a total of 1369 patients with OCRD were enrolled. The mean (SD) age was 31.5 (7.8) years, 65.6% were female (847 of 1292), and 34.4% were male (445 of 1292). Most studies (17 of 27) had a low risk of bias,^36,38,41,43,44,45,46,47,48,50,53,54,55,58,59,60,61^ 7 had some concerns of bias,^37,40,42,51,56,57,62^ and 3 studies were categorized as having a high risk of bias.^39,49,52^ A detailed bias assessment can be found in eFigures 1 and 2 in Supplement 1. The geographic distribution of the studies was as follows: Iran (n = 15), US (n = 8), Australia (n = 2), Brazil (n = 1), and Italy (n = 1). Four studies included children and adolescents, while the remaining 23 included adults. Obsessive-compulsive disorder encompassed most of the studies (n = 23), complemented by 2 studies each on trichotillomania and skin-picking disorder. Regarding treatment modalities, 10 studies investigated NAC, 4 explored memantine, 3 each assessed lamotrigine and riluzole, 2 examined topiramate, and 1 study each assessed amantadine, glycine, l-carnosine, minocycline, and pregabalin. All studies reported minimal or no adverse effects. Among those that did, the adverse effects were generally mild to moderate, including nausea and vomiting, diarrhea, stomach and abdominal pain, and heartburn—all with NAC. The Table provides further details of the included studies.^36,37,38,39,40,41,42,43,44,45,46,47,48,49,50,51,52,53,54,55,56,57,58,59,60,61,62^

**Table.** Characteristics of Included Studies

Source	Country	Sample size	Population	Type of OCRD	Refractoriness	Age, mean (SD), y	Sex, male, No. (%)	Years With OCRD, mean (SD)	Comorbid psychiatric disorders	Medication	Dose duration	Augmentation strategy	Control	Outcome	Significant frequency of adverse effects
Afshar et al,^36^ 2012	Iran	I: 24 C: 24	Adults	OCD	Yes, as defined by Y-BOCS score ≥16 after ≥12 wk of treatment with SSRI	I: 30.6 (5.4) C: 31.3 (4.7)	I: 6 (25) C: 6 (25)	I: 15.5 (8.6) C: 16.4 (9.4)	NR	NAC	Initial dosage of 600 mg/d, increased weekly to a maximum of 2400 mg/d over 12 wk	Not randomized to SSRI; treatment with SSRI continued throughout the study with the same dose	Placebo	Y-BOCS total score	Mild to moderate nausea and vomiting, and mild diarrhea in the treatment group
Afshar et al,^37^ 2014	Iran	I: 19 C: 19	Adults	OCD	Yes, as defined by Y-BOCS score ≥16 after ≥12 wk of treatment with SSRI	I: 34.8 (10.7) C: 33.4 (8.6)^a^	I: 1 (6.3) C: 1 (6.7)^a^	I: 9.5 (5.5) C: 8.4 (5.5)^a^	NR	Topiramate	Initial dosage of 25 mg/d, increased weekly to a maximum of 200 mg/d over 12 wk	Not randomized to SSRI; treatment with SSRI continued throughout the study with the same dose	Placebo	Y-BOCS total score	No
Arabzadeh et al,^38^ 2017	Iran	I: 25 C: 25	Adults	OCD	No	I: 33.5 (13.8) C: 30.5 (8.3)^a^	I: 8 (36.4) C: 10 (45.5)^a^	I: 6.6 (8.5) C: 5.6 (6.2)^a^	Excluded major psychiatric disorders^b^	l-Carnosine	500 mg twice daily over 10 wk	Fluvoxamine 100 mg/d for 4 wk, increased to 200 mg/d for the following 6 wk	Placebo and fluvoxamine	Y-BOCS total score	No
Askari et al,^39^ 2022	Iran	I: 35 C: 35	Adults	OCD	No	I: 35 (11.4) C: 33.8 (10.3)^a^	I: 8 (22.9) C: 13 (43.3)^a^	NR	Excluded axis I and other disorders^c^	Memantine	Initial dosage of 5 mg/d, increased slowly to a maximum dosage of 10 mg twice daily over 12 wk	Sertraline 100 mg/d for 4 wk (initial dosage with 25 mg/d), increased gradually to 200 mg/d for the following 8 wk	Placebo and sertraline	Y-BOCS total score	No
Bloch et al,^40^ 2013	US	I: 20 C: 19	Children and adolescents	TTM	No	I: 14 (2.4) C: 13.1 (3.1)	I: 3 (15) C: 2 (11)	I: 4.4 (2.6) C: 3.3 (2.4)	MDD, anxiety disorder, OCD, SPD, tic disorder, and ADHD^c^	NAC	Initial dosage of 600 mg/d, increased weekly to a maximum dosage of 1200 mg twice daily over 12 wk	Not randomized to SSRI; stable medication allowed	Placebo	MGH-HPS total score	No
Bruno et al,^41^ 2012	Italy	I: 20 C: 20	Adults	OCD	Yes, as defined by Y-BOCS score ≥16 after ≥12 wk of treatment with SSRI	I: 34.2 (10.3) C: 38.5 (11.3)	I: 7 (35) C: 9 (45)	I: 6.1 (2.3) C: 5.8 (2.6)	No	Lamotrigine	Initial dosage of 25 mg/d, increased weekly to a maximum dosage of 100 mg/d over 16 wk	Not randomized to SSRI; doses of SSRI needed to be stable for ≥2 mo and left unchanged during the study	Placebo	Y-BOCS total score	No
Costa et al,^42^ 2017	Brazil	I: 18 C: 22	Adults	OCD	Yes, as defined by Y-BOCS score ≥16 after ≥12 wk of treatment with SSRI or clomipramine	I: 37.8 (10.5) C: 38.2 (11.3)	I: 8 (44.4) C: 13 (59.1)	Age at onset: I: 11.8 (6) C: 11.7 (5.2)	MDD and anxiety disorder^c^	NAC	Initial dosage of 600 mg twice daily, increased weekly to a maximum dosage of 3000 mg/d over 16 wk	Not randomized to SSRI; SSRI under a stable dose allowed	Placebo	Y-BOCS total score	Stomach and abdominal pain in the treatment group
Emamzadehfard et al, 2016^43^	Iran	I: 27 C: 27	Adults	OCD	No	I: 33.7 (10.5) C: 36 (10)^a^	I: 9 (36) C: 6 (24)^a^	I: 7.2 (4) C: 5.9 (3.1)^a^	Excluded axis I and other disorders^c^	Riluzole	50 mg twice daily over 10 wk	Fluvoxamine 100 mg/d for 4 wk, increased to 200 mg/d for the following 6 wk	Placebo and fluvoxamine	Y-BOCS total score	No
Esalatmanesh et al,^44^ 2016	Iran	I: 51 C: 51	Adults	OCD	No	I: 34.7 (7.9) C: 34 (8.9)^a^	I: 18 (38.3) C: 18 (38.3)^a^	I: 4.5 (2.6) C: 5.3 (2.9)^a^	Excluded axis I and other disorders^c^	Minocycline	100 mg twice daily over 10 wk	Fluvoxamine 100 mg/d for 4 wk, increased to 200 mg/d for the following 6 wk	Placebo and fluvoxamine	Y-BOCS total score	No
Ghaleiha et al,^45^ 2013	Iran	I: 21 C: 21	Adults	OCD	No	I: 36.2 (6) C: 37.5 (6.2)^a^	I: 7 (35) C: 5 (26)^a^	I: 5.3 (2.6) C: 5.9 (2.9)^a^	Excluded axis I and other disorders^c^	Memantine	Initial dosage of 10 mg/d in first week, increased to 20 mg/d in second week, and maintained dosage for following 6 wk	Fluvoxamine 100 mg/d for 4 wk, increased to 200 mg/d for the following 4 wk	Placebo and fluvoxamine	Y-BOCS total score	No
Ghanizadeh et al,^46^ 2017	Iran	I: 19 C: 15	Children and adolescents	OCD	No	I: 16.5 (2.9) C: 15.9 (3.7)^a^	I: 11 (61.1) C: 4 (36.2)^a^	NR	Excluded some comorbidities^c^	NAC	Initial dosage of 600 mg/d, increased weekly to a maximum dosage of 2400 mg/d over 10 wk	Citalopram 20-40 mg/d	Placebo and citalopram	Y-BOCS total score	No
Grant et al,^47^ 2009	US	I: 25 C: 25	Adults	TTM	No	I: 32.7 (10.5) C: 35.8 (13.6)	I: 1 (4) C: 4 (16)	Age at onset: I: 11.2 (4.7) C: 15.1 (8.5)	MDD, GAD, anxiety disorder, PTSD, anxiety disorder not specified, OCD, SPD, compulsive nail biting, and eating disorder^c^	NAC	Initial dosage of 1200 mg/d during first 6 wk, increased to 2400 mg/d for following 6 wk	Not randomized to SSRI; SSRI needed to be stable for 3 mo	Placebo	MGH-HPS total score	No
Grant et al,^48^ 2010	US	I: 16 C: 16	Adults	SPD	No	I: 33.2 (14.1) C: 31.6 (13.3)	I: 1 (6.3) C: 2 (12.5)	Age at onset: 13 (9.2) for the entire cohort	No	Lamotrigine	Initial dosage of 25 mg every other day, increased weekly to a maximum dosage of 300 mg/d over 12 wk	Not randomized to SSRI; use of psychotropic medication was an exclusion criteria	Placebo	NE-YBOCS total score	No
Grant et al,^49^ 2016	US	I: 35 C: 31	Adults	SPD	No	I: 34.9 (11.6) C: 34.7 (10.5)	I: 2 (6) C: 6 (19)	Age at onset: I: 11.7 (6.9) C: 12.8 (6)	MDD, anxiety disorder, TTM, and compulsive nail biting^b^	NAC	Initial dosage of 1200 mg/d, increased to 2400 mg/d by week 3, and then increased to 3000 mg/d by week 6 for remaining 6 wk	Not randomized to SSRI; stable dose of SSRI for 3 mo allowed	Placebo	NE-YBOCS total score	No
Grant et al,^50^ 2014	US	I: 30 C: 30	Children and adolescents	OCD	Yes, as defined by Y-BOCS score ≥20 and treatment resistant	I: 14.8 (2.1) C: 14.2 (2.6)	I: 22 (73) C: 22 (73)	NR	ASD^c^	Riluzole	Initial dosage of 10 mg/d, increased daily to a maximum dosage of 100 mg/d over 12 wk	Not randomized to SSRI; use of psychotropic medication allowed	Placebo	CY-BOCS total score	No
Greenberg et al,^51^ 2009	US	I: 12 C: 12	Adults	OCD	No	I: 44.2 (14.3) C: 36.1 (12.2)	I: 5 (41.6) C: 4 (33.3)	NR	MDD and anxiety disorder^c^	Glycine	30 g twice daily over 12 wk	Not randomized to SSRI; stable dose of SSRI for 3 mo allowed	Placebo	Y-BOCS total score	NR
Haghighi et al,^52^ 2013	Iran	I: 20 C: 20	Adults	OCD	Yes, Y-BOCS score ≥21 after ≥12 wk of treatment with SSRI	I: 30.8 (6) C: 31.6 (5.1)^a^	I: 2 (14.3) C: 4 (26.7)^a^	I: 4.1 (1.9) C: 3.8 (1.5)^a^	No	Memantine	Initial dosage of 5 mg/d, increased to 10 mg/d over 12 wk	Not randomized to SSRI; stable dose of SSRI for 3 mo allowed	Placebo	Y-BOCS total score	NR
Khalkhali et al,^53^ 2016	Iran	I: 30 C: 30	Adults	OCD	Yes, as defined by Y-BOCS score ≥21 after ≥12 wk with 2 different SSRIs	<31: I: 30% C: 23.3% 31-40: I: 43.3% C: 40% >40: I: 26.7% C: 36.7%	I: 7 (23.3) C: 10 (33.3)	1-3 y: I: 13.3% C: 20% 3-5 y: I: 26.7% C: 13.3% >5 y: I: 60% C: 66.7%	Excluded major psychiatric disorders^c^	Lamotrigine	Initial dosage of 25 mg/d, increased weekly to a maximum dosage of 100 mg/d over 12 wk	Not randomized to SSRI; stable dose of SSRI for 3 mo allowed	Placebo	Y-BOCS total score	No
Li et al,^54^ 2020	US	I: 5 C: 6	Children and adolescents	OCD	No	I: 13.4 (3.4) C: 10.7 (1.9)	I: 2 (40) C: 1 (16.7)	NR	MDD, anxiety disorder, PTSD, Tourette syndrome, and ADHD^b^	NAC	Initial dosage of 900 mg/d, increased weekly to a maximum dosage of 900 mg 3 times daily over 12 wk	Not randomized to SSRI; stable dose of SSRI in the last 12 mo allowed	Placebo	CY-BOCS total score	No
Modarresi et al,^55^ 2018	Iran	I: 16 C: 16	Adults	OCD	Yes, as defined by Y-BOCS score ≥24 after ≥3 adequate trials of SSRI therapy, 1 of which was clomipramine	I: 30.6 (6.8) C: 30.7 (4.7)^a^	I: 6 (40) C: 5 (33)^a^	I: 1.7 (0.4) C: 1.6 (0.5)^a^	Excluded several comorbid psychiatric disorders^b^	Memantine	10 mg twice daily over 12 wk	Not randomized to SSRI; stable dose of SSRI for 3 mo allowed	Placebo	Y-BOCS total score	No
Mowla et al,^56^ 2010	Iran	I: 24 C: 25	Adults	OCD	Yes, as defined by Y-BOCS score ≥18 after ≥12 wk of treatment with SSRI	I: 35.4 (NR) C: 33.2 (NR)	I: 10 (41.7) C: 11 (44.5)	I: 7.5 (NR) C: 8.7 (NR)	Excluded axis I and II disorders^c^	Topiramate	Initial dosage of 25 mg/d, increased weekly to a maximum dosage of 200 mg/d over 12 wk	Not randomized to SSRI; previous use of SSRI allowed	Placebo	Y-BOCS total score	NR
Mowla et al,^57^ 2020	Iran	I: 28 C: 28	Adults	OCD	Yes, as defined by Y-BOCS score ≥18 after ≥12 wk of treatment with SSRI	I: 33.6 (11.3) C: 31.1 (12.2)^a^	I: 8 (34.6) C: 7 (32.8)^a^	NR	No	Pregabalin	Initial dosage of 75 mg/d, increased weekly to a maximum dosage of 225 mg/d over 12 wk	Sertraline 100-300 mg/d depending on the patient’s intolerance	Placebo and sertraline	Y-BOCS total score	No
Naderi et al,^58^ 2019	Iran	I: 53 C: 53	Adults	OCD	No	I: 34.8 (9.2) C: 34.4 (8.6)^a^	I: 23 (45.1) C: 23 (46.9)^a^	I: 4.6 (2.5) C: 5.2 (2.9)^a^	Excluded axis I disorders^c^	Amantadine	100 mg/d over 12 wk	Fluvoxamine 100 mg twice daily over 12 wk	Placebo and fluvoxamine	Y-BOCS total score	No
Paydary et al,^59^ 2016	Iran	I: 23 C: 23	Adults	OCD	No	I: 33.7 (11.3) C: 33 (11.4)^a^	I: 5 (22.8) C: 6 (27.3)^a^	I: 6.9 (4) C: 5.8 (4.5)^a^	Excluded axis I disorders^c^	NAC	Initial dosage of 500 mg twice daily for first week, increased to 1000 mg twice daily for following 9 wk	Fluvoxamine 100 mg/d for the first 4 wk, increased to 200 mg/d for the following 6 wk	Placebo and fluvoxamine	Y-BOCS total score	No
Pittenger et al,^60^ 2015	US	I: 20 C: 18	Adults	OCD	Yes, as defined by failure of ≥1 previous adequate dose of SSRI	I: 41.5 (13.9) C: 36.4 (13.2)^a^	I: 11 (57.9) C: 9 (50)^a^	Age at onset: I: 27.8 (3.6) C: 19.5 (2.3)^a^	Anxiety disorders, MDD, and tics^c^	Riluzole	50 mg twice daily over 12 wk	Not randomized to SSRI; stable dose with SSRI or clomipramine at a stable effective dose for >8 wk allowed	Placebo	Y-BOCS total score	No
Sarris et al,^61^ 2015	Australia	I: 22 C: 22	Adults	OCD	No	I: 39.1 (12.8) C: 34.9 (11.4)	I: 12 (54.5) C: 12 (54.5)	I: 17.6 (8.9) C: 15.1 (10.1)	Yes, but did not specify^c^	NAC	Initial dosage of 500 mg twice daily, increased weekly to a maximum dosage of 1500 mg twice daily over 16 wk	Not randomized to SSRI; stable treatment allowed	Placebo	Y-BOCS total score	Heartburn in the treatment group
Sarris et al,^62^ 2022	Australia	I: 50 C: 48	Adults	OCD	No	Median (IQR): I: 31.5 (20.8) C: 32.0 (21.0)^a^	I: 10 (22.7) C: 19 (42.2)^a^	Median (IQR): I: 9 (14) C: 6 (15)^a^	MDD and GAD^b^	NAC	Initial dosage of 1000 mg twice daily in first 8 wk, increased to a maximum dosage of 2000 mg twice daily in cases of nonresponse for following 12 wk	Not randomized to SSRI; stable treatment allowed	Placebo	Y-BOCS total score	No

Abbreviations: ADHD, attention-deficit/hyperactivity disorder; ASD, autism spectrum disorder; C, control group; CY-BOCS, Children’s Yale-Brown Obsessive Compulsive Scale; GAD, generalized anxiety disorder; I, intervention group; MDD, major depressive disorder; MGH-HPS, Massachusetts General Hospital Hairpulling Scale; NAC, *N*-acetylcysteine; NE-YBOCS, Yale-Brown Obsessive Compulsive Scale Modified for neurotic excoriation; NR, not reported; OCD, obsessive-compulsive disorder; OCRD, obsessive-compulsive and related disorders; PTSD, posttraumatic stress disorder; SPD, skin picking disorder; SSRI, selective serotonin reuptake inhibitor; TTM, trichotillomania; Y-BOCS, Yale-Brown Obsessive Compulsive Scale.

^a^ Based on the number at baseline, which may differ from the total number enrolled. This discrepancy occurs because some studies only reported baseline data for analyzed participants rather than the full randomized sample.

^b^ Based on the *Diagnostic and Statistical Manual of Mental Disorders* (Fifth Edition).

^c^ Based on the *Diagnostic and Statistical Manual of Mental Disorders* (Fourth Edition).

### OCRD Analysis

**Figure 2.**
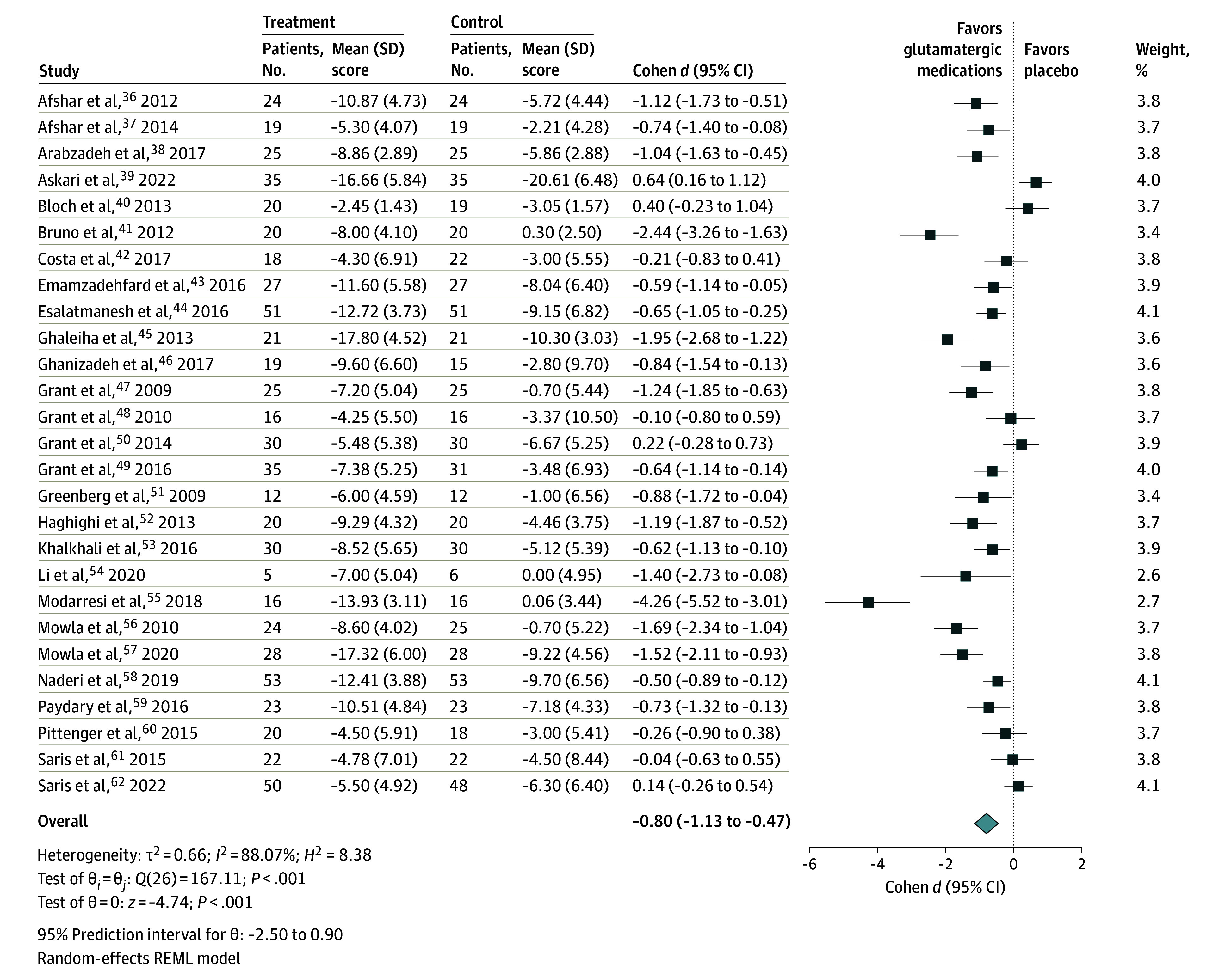
Forest Plot of Glutamatergic Medications for Symptoms of Obsessive-Compulsive and Related Disorders The mean score depends on the scale being used, as it varies across the different disorders within the obsessive-compulsive and related disorders spectrum (see Methods). REML indicates restricted maximum likelihood.

A total of 27 studies^36,37,38,39,40,41,42,43,44,45,46,47,48,49,50,51,52,53,54,55,56,57,58,59,60,61,62^ were included in the meta-analysis to evaluate glutamatergic medications for OCRD symptoms. There was a large effect size (Cohen *d* = −0.80 [95% CI, −1.13 to −0.47]; *P* < .001; *I*^2^ = 88%) when comparing the intervention group (n = 688) with the control group (n = 681) (Figure 2),^36,37,38,39,40,41,42,43,44,45,46,47,48,49,50,51,52,53,54,55,56,57,58,59,60,61,62^ with evidence of publication bias (Egger test, *P* < .001) (eFigure 3 in Supplement 1). The level of certainty was categorized as low (eTable 7 in Supplement 1).

Subgroup analyses indicated no significant differences in the magnitude of the effect size of glutamatergic medications on OCRD symptoms by type of OCRD, population, refractoriness of OCRD, augmentation strategy, risk of bias, or type of glutamatergic medication. Detailed results of the subgroup analyses are provided in eFigures 4 to 9 in Supplement 1. Sensitivity analysis with a leave-one-out analysis showed that the exclusion of any single study did not significantly alter the overall effect size (eFigure 10 in Supplement 1). Univariate meta-regression analyses found no statistically significant estimates when controlling for mean age, mean years living with OCRD, or weeks of treatment (eTable 8 in Supplement 1).

### OCD Analysis

**Figure 3.**
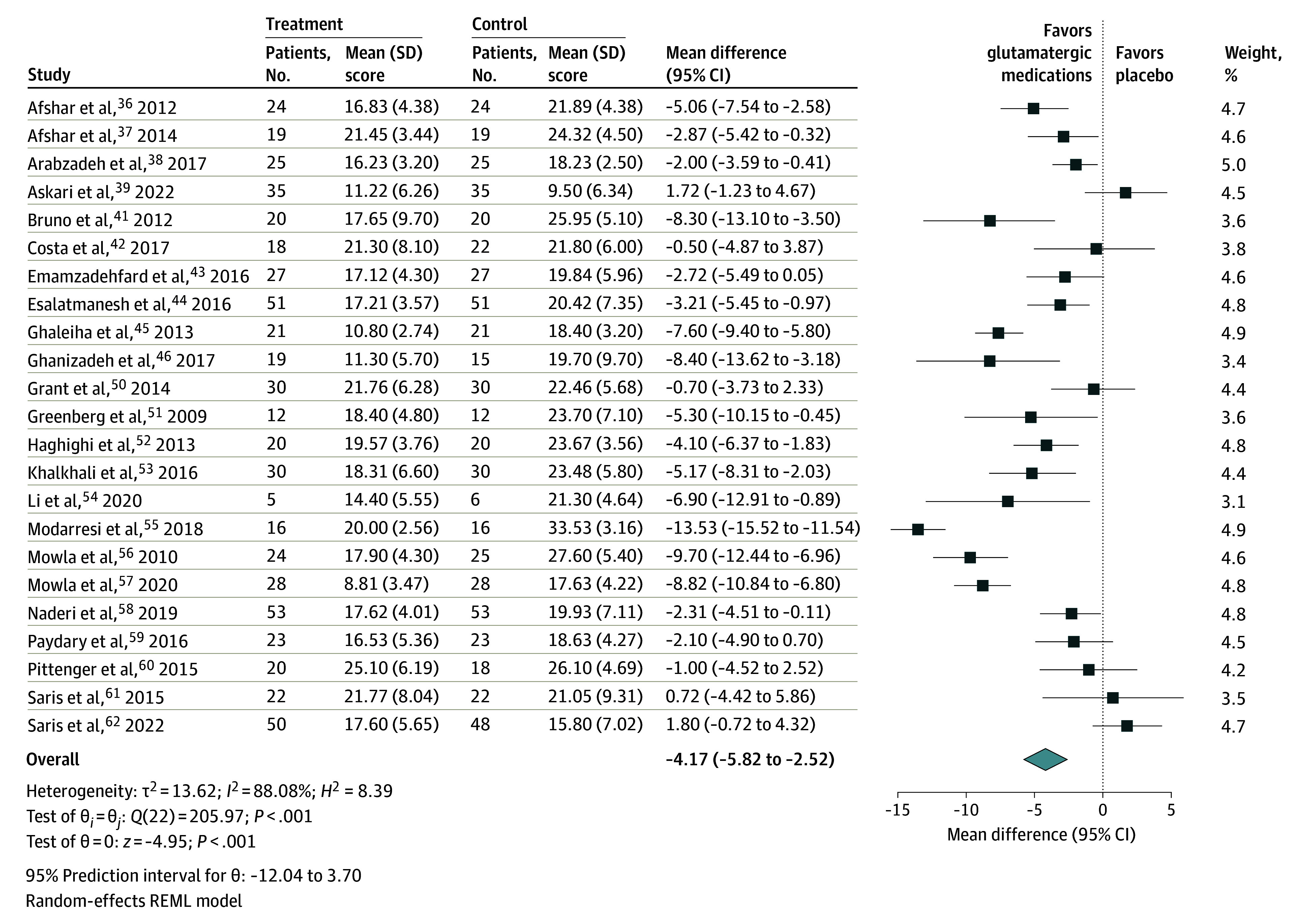
Forest Plot of Glutamatergic Medications for Symptoms of Obsessive-Compulsive Disorder The Yale-Brown Obsessive Compulsive Scale was used for obsessive-compulsive symptoms and is a clinician-administered scale that assesses the severity of obsessive-compulsive symptoms, consisting of 10 items—5 for obsessions and 5 for compulsions—with total scores ranging from 0 to 40, where higher scores indicate greater symptom severity. REML indicates restricted maximum likelihood.

A total of 23 studies^36,37,38,39,41,42,43,44,45,46,50,51,52,53,54,55,56,57,58,59,60,61,62^ were included in the meta-analysis to evaluate glutamatergic medications for OCD symptoms. There was a statistically significant mean reduction in Y-BOCS scores (mean difference, −4.17 [95% CI, −5.82 to −2.52]; *P* < .001; *I*^2^ = 88%) when comparing the intervention group (n = 592) with the control group (n = 590) (Figure 3),^36,37,38,39,41,42,43,44,45,46,50,51,52,53,54,55,56,57,58,59,60,61,62^ with no evidence of publication bias (Egger test, *P* = .84) (eFigure 11 in Supplement 1). The level of certainty was categorized as moderate (eTable 7 in Supplement 1).

Subgroup analyses indicated no significant mean differences by population, refractoriness of OCD, augmentation strategy, risk of bias, or type of glutamatergic medication. Detailed results of the subgroup analyses are provided in eFigures 12 to 16 in Supplement 1. Sensitivity analysis with a leave-one-out analysis showed that the exclusion of any single study did not significantly alter the overall mean difference (eFigure 17 in Supplement 1). Univariate meta-regression analyses found no statistically significant estimates when controlling for mean age, mean years living with OCD, or weeks of treatment (eTable 9 in Supplement 1).

## Discussion

This systematic review and meta-analysis evaluated glutamatergic medications to treat symptoms of OCRDs. Our findings, albeit with low certainty, indicate that these medications are associated with significant improvement in OCRD symptoms. When restricting to OCD trials, our evidence suggests, with moderate certainty, that these medications are associated with significantly reduced Y-BOCS scores. In the subgroup analyses for both OCRDs and OCD, no significant differences were observed across subgroups.

Our results align with the emerging understanding of the role of the glutamatergic system in the pathophysiology of OCRDs.^16,17^ Glutamatergic medications can modulate synaptic plasticity and neuronal excitability, potentially alleviating OCRD symptoms.^17^ For instance, NAC has been shown to increase glutathione levels and modulate the glutamatergic system, reducing oxidative stress.^63^ Memantine, an *N*-methyl-d-aspartate receptor antagonist, has been demonstrated to decrease excessive glutamate transmission in the corticostriatal pathways.^64^ Lamotrigine may stabilize neuronal membranes and inhibit glutamate release,^65^ while topiramate might reduce neuronal hyperactivity through its inhibitory effects on the alpha-amino-3-hydroxy-5-methyl-4-isoxazole propionic acid and kainate subtypes of glutamate receptors.^66^ These alterations in the glutamatergic system might be associated with symptom improvement, particularly in OCD, as evidenced by our analysis.

Further subgroup analyses for OCRDs and OCD did not reveal statistically significant differences, indicating no variation by type of OCRD, population, refractoriness of OCRD or OCD, augmentation strategy, risk of bias, or type of glutamatergic medication. The analysis regarding augmentation strategy presents a more complex scenario. Although no significant differences were found, many studies allowed participants to continue their standard treatments, often including SSRIs. This confounding factor limits our ability to draw definitive conclusions about this specific subgroup analysis.

The high heterogeneity and indications of publication bias identified in our study warrant a cautious interpretation of the findings. This variability likely reflects the diverse range of disorders, patient populations, and treatment approaches within the OCRD spectrum. The asymmetry observed in the funnel plot points to the possibility of small study effects or some degree of publication bias. However, in more homogeneous subgroups, such as those treated with medications such as topiramate or riluzole, we observed reduced heterogeneity, indicating that targeted treatments may yield more consistent and predictable outcomes. In addition, our sensitivity analyses, including leave-one-out analysis, consistently demonstrated the association of glutamatergic medications with symptom improvement. This consistency across analyses suggests that the observed positive associations are robust and not driven by any single study. Furthermore, most of the trials included in this review were categorized as low risk of bias, adding to the reliability of the results.

This systematic review differs from prior reviews by focusing on a broader population of OCRDs. Previous reviews often targeted specific groups, such as individuals with OCD, or specific glutamatergic medications, such as NAC and memantine.^21,22,23,24,25^ Our analysis extends these insights by offering a more comprehensive evaluation across a wider spectrum of OCRDs and focusing on subgroup analyses based on important clinical characteristics.

### Limitations

This systematic review and meta-analysis has some limitations, including the relatively small sample sizes of the trials and the limited number of studies for some subgroups, such as those testing lamotrigine and topiramate. In addition, we excluded non-English studies and we did not search the gray literature. We were also unable to assess dose-dependent effects due to dose escalation and inconsistent reporting across the included studies, and a network meta-analysis was beyond the scope of our review. Moreover, the review included only 2 studies each for disorders such as skin picking disorder and trichotillomania, and no studies met the criteria for other OCRDs, such as BDD and hoarding disorder. Furthermore, promising therapeutic glutamatergic medications, such as ketamine and troriluzole, did not meet our inclusion criteria and warrant further research.

## Conclusions

This systematic review and meta-analysis indicates that glutamatergic medications may be effective in treating OCRDs, particularly OCD. However, high heterogeneity and potential publication bias necessitate cautious interpretation. Future research with larger sample sizes should focus on dose-dependent effects, additional OCRD subtypes, and novel glutamatergic treatments to enhance our understanding and treatment strategies.

## Notes

Supplement 1. **eTable 1.** Full Search Strategy for PubMed **eTable 2.** Full Search Strategy for Embase **eTable 3.** Full Search Strategy for PsycINFO **eTable 4.** Full Search Strategy for Web of Science **eTable 5.** Full Search Strategy for Cochrane Central Register of Controlled Trials **eTable 6.** List of Excluded Studies **eFigure 1.** Cochrane Risk of Bias for Randomized Controlled Trials by Domains **eFigure 2.** Cochrane Risk of Bias for Randomized Controlled Trials Overall **eTable 7.** Summary of GRADE **eFigure 3.** Meta-Analysis Obsessive-Compulsive and Related Disorders (OCRDs): Funnel Plot **eFigure 4.** Subgroup Analysis Based on Type of OCRD **eFigure 5.** Subgroup Analysis Based on Population **eFigure 6.** Subgroup Analysis Based on Refractoriness of OCRD **eFigure 7.** Subgroup Analysis Based on Augmentation Strategy **eFigure 8.** Subgroup Analysis Based on Risk of Bias **eFigure 9.** Subgroup Analysis Based on Type of Glutamatergic Medication **eFigure 10.** Sensitivity Analysis Using a Leave-One-Out Analysis **eTable 8.** Univariate Meta-Regression by Mean Age, Mean Years Living With OCRD, or Weeks of Treatment **eFigure 11.** Meta-Analysis Obsessive-Compulsive Disorder (OCD): Funnel Plot **eFigure 12.** Subgroup Analysis Based on Population **eFigure 13.** Subgroup Analysis Based on Refractoriness of OCD **eFigure 14.** Subgroup Analysis Based on Augmentation Strategy **eFigure 15.** Subgroup Analysis Based on Risk of Bias **eFigure 16.** Subgroup Analysis Based on Type of Glutamatergic Medication **eFigure 17.** Sensitivity Analysis Using a Leave-One-Out Analysis **eTable 9.** Univariate Meta-Regression by Mean Age, Mean Years Living With OCD, or Weeks of Treatment

Supplement 2.
Data Sharing Statement
