# Supplementary material for: Glutamatergic Medications for Obsessive-Compulsive and Related Disorders: A Systematic Review and Meta-Analysis
Source: JAMA Netw Open. 2025 Jan 2;8(1):e2452963. doi: 10.1001/jamanetworkopen.2024.52963 (PMC11696454; doi:10.1001/jamanetworkopen.2024.52963)
Supplement: Supplement 1. — eTable 1. Full Search Strategy for PubMed eTable 2. Full Search Strategy for Embase eTable 3. Full Search Strategy for PsycINFO eTable 4. Full Search Strategy for Web of Science eTable 5. Full Search Strategy for Cochrane Central Register of Controlled Trials eTable 6. List of Excluded Studies eFigure 1. Cochrane Risk of Bias for Randomized Controlled Trials by Domains eFigure 2. Cochrane Risk of Bias for Randomized Controlled Trials Overall eTable 7. Summary of GRADE eFigure 3. Meta-Analysis Obsessive-Compulsive and Related Disorders (OCRDs): Funnel Plot eFigure 4. Subgroup Analysis Based on Type of OCRD eFigure 5. Subgroup Analysis Based on Population eFigure 6. Subgroup Analysis Based on Refractoriness of OCRD eFigure 7. Subgroup Analysis Based on Augmentation Strategy eFigure 8. Subgroup Analysis Based on Risk of Bias eFigure 9. Subgroup Analysis Based on Type of Glutamatergic Medication eFigure 10. Sensitivity Analysis Using a Leave-One-Out Analysis eTable 8. Univariate Meta-Regression by Mean Age, Mean Years Living With OCRD, or Weeks of Treatment eFigure 11. Meta-Analysis Obsessive-Compulsive Disorder (OCD): Funnel Plot eFigure 12. Subgroup Analysis Based on Population eFigure 13. Subgroup Analysis Based on Refractoriness of OCD eFigure 14. Subgroup Analysis Based on Augmentation Strategy eFigure 15. Subgroup Analysis Based on Risk of Bias eFigure 16. Subgroup Analysis Based on Type of Glutamatergic Medication eFigure 17. Sensitivity Analysis Using a Leave-One-Out Analysis eTable 9. Univariate Meta-Regression by Mean Age, Mean Years Living With OCD, or Weeks of Treatment [file jamanetwopen-e2452963-s001.pdf]

Supplementary Online Content

Coelho DRA, Yang C, Suriaga A, et al. Glutamatergic medications for obsessive-compulsive and related disorders: a systematic review and meta-analysis. *JAMA Netw Open.* 2025;8(1):e2452963. doi:10.1001/jamanetworkopen.2024.52963

eTable 1. Full Search Strategy for PubMed
eTable 2. Full Search Strategy for Embase
eTable 3. Full Search Strategy for PsycINFO
eTable 4. Full Search Strategy for Web of Science
eTable 5. Full Search Strategy for Cochrane Central Register of Controlled Trials
eTable 6. List of Excluded Studies
eFigure 1. Cochrane Risk of Bias for Randomized Controlled Trials by Domains
eFigure 2. Cochrane Risk of Bias for Randomized Controlled Trials Overall
eTable 7. Summary of GRADE
eFigure 3. Meta-Analysis Obsessive-Compulsive and Related Disorders (OCRDs): Funnel Plot
eFigure 4. Subgroup Analysis Based on Type of OCRD
eFigure 5. Subgroup Analysis Based on Population
eFigure 6. Subgroup Analysis Based on Refractoriness of OCRD
eFigure 7. Subgroup Analysis Based on Augmentation Strategy
eFigure 8. Subgroup Analysis Based on Risk of Bias
eFigure 9. Subgroup Analysis Based on Type of Glutamatergic Medication
eFigure 10. Sensitivity Analysis Using a Leave-One-Out Analysis
eTable 8. Univariate Meta-Regression by Mean Age, Mean Years Living With OCRD, or Weeks of Treatment
eFigure 11. Meta-Analysis Obsessive-Compulsive Disorder (OCD): Funnel Plot
eFigure 12. Subgroup Analysis Based on Population
eFigure 13. Subgroup Analysis Based on Refractoriness of OCD
eFigure 14. Subgroup Analysis Based on Augmentation Strategy
eFigure 15. Subgroup Analysis Based on Risk of Bias
eFigure 16. Subgroup Analysis Based on Type of Glutamatergic Medication
eFigure 17. Sensitivity Analysis Using a Leave-One-Out Analysis
eTable 9. Univariate Meta-Regression by Mean Age, Mean Years Living With OCD, or Weeks of Treatment

This supplementary material has been provided by the authors to give readers additional information about their work.

© 2025 Coelho DRA et al. *JAMA Network Open.*

eTable 1. Full Search Strategy for PubMed

1	OCRD[tiab] OR "Obsessive-Compulsive Disorder"[Mesh] OR OCD[tiab] OR obsess*[tiab] OR compulsi*[tiab] OR "Body Dysmorphic Disorders"[Mesh] OR "body dysmorphic disorder"[tiab] OR BDD[tiab] OR "image disorder"[tiab] OR "image dysfunction"[tiab] OR Trichotillomania[Mesh] OR trichotillomania[tiab] OR "hair-pulling"[tiab] OR trichobezoar[tiab] OR excoriation[tiab] OR "skin-picking"[tiab] OR hoarding[tiab] OR "pathological collecting"[tiab] OR "hoarder"[tiab]
2	"Excitatory Amino Acid Agents"[Mesh] OR glutamat*[tiab] OR NMDA[tiab] OR AMPA[tiab] OR Lamotrigine[Mesh] OR lamotrigin*[tiab] OR Topiramate[Mesh] OR topiramat*[tiab] OR Gabapentin[Mesh] OR gabapentin*[tiab] OR Pregabalin[Mesh] OR pregabalin*[tiab] OR Memantine[Mesh] OR memantin*[tiab] OR Amantadine[Mesh] OR amantadin*[tiab] OR Ketamine[Mesh] OR ketamin*[tiab] OR Esketamine[Supplementary Concept] OR esketamin*[tiab] OR Dextromethorphan[Mesh] OR dextromethorphan*[tiab] OR Agmatine[Mesh] OR agmatin*[tiab] OR Minocycline[Mesh] OR minocyclin*[tiab] OR Acetylcysteine[Mesh] OR acetylcystein*[tiab] OR n-acetylcystein*[tiab] OR NAC[tiab] OR "Glycine Agents"[Mesh] OR glycin*[tiab] OR Cycloserine[Mesh] OR cycloserin*[tiab] OR d-cycloserin*[tiab] OR Sarcosine[Mesh] OR sarcosin*[tiab] OR Carnosine[Mesh] OR carnosin*[tiab] OR L-carnosin*[tiab] OR Riluzole[Mesh] OR riluzol*[tiab] OR Modafinil[Mesh] OR modafinil*[tiab]
3	"Randomized Controlled Trials as Topic"[Mesh] OR "Randomized Controlled Trial"[Pt] OR "Controlled Clinical Trial"[Pt] OR "Clinical trial"[Pt] OR randomized[tiab] OR randomly[tiab] OR trial[tiab] OR placebo[tiab]
4	#1 AND #2 AND #3

© 2025 Coelho DRA et al. *JAMA Network Open.*

eTable 2. Full Search Strategy for Embase

1	'obsessive compulsive disorder'/exp OR 'body dysmorphic disorder'/exp OR 'trichotillomania'/exp OR 'skin picking disorder'/exp OR 'hoarding disorder'/exp OR (OCRD OR OCD OR obsess* OR compulsi* OR 'body dysmorphic disorder' OR BDD OR 'image disorder' OR 'image dysfunction' OR trichotillomania OR 'hair-pulling' OR trichobezoar OR excoriation OR 'skin-picking' OR hoarding OR 'pathological collecting' OR hoarder):ab,kw,ti
2	'glutamatergic signaling'/exp OR 'lamotrigine'/exp OR 'topiramate'/exp OR 'gabapentin'/exp OR 'pregabalin'/exp OR 'memantine'/exp OR 'amantadine'/exp OR 'ketamine'/exp OR 'esketamine'/exp OR 'dextromethorphan'/exp OR 'agmatine'/exp OR 'minocycline'/exp OR 'acetylcysteine'/exp OR 'glycine'/exp OR 'cycloserine'/exp OR 'sarcosine'/exp OR 'carnosine'/exp OR 'riluzole'/exp OR 'modafinil'/exp OR (glutamat* OR NMDA OR AMPA OR lamotrigin* OR topiramat* OR gabapentin* OR pregabalin* OR memantin* OR amantadin* OR ketamin* OR esketamin* OR dextromethorphan* OR agmatin* OR minocyclin* OR acetylcystein* OR n-acetylcystein* OR NAC OR glycin* OR cycloserin* OR d-cycloserin* OR sarcosin* OR carnosin* OR L-carnosin* OR riluzol* OR modafinil*):ab,kw,ti
3	'controlled clinical trial'/exp OR (random* OR trial OR placebo):ab,kw,ti
4	#1 AND #2 AND #3 [embase]/lim
5	#4 NOT 'conference abstract'/it

© 2025 Coelho DRA et al. *JAMA Network Open.*

eTable 3. Full Search Strategy for PsycINFO

1	OCRD OR OCD OR obsess* OR compulsi* OR "body dysmorphic disorder" OR BDD OR "image disorder" OR "image dysfunction" OR trichotillomania OR "hair-pulling" OR trichobezoar OR excoriation OR "skin-picking" OR hoarding OR "pathological collecting" OR hoarder
2	"excitatory amino acid agents" OR glutamat* OR NMDA OR AMPA OR lamotrigin* OR topiramat* OR gabapentin* OR pregabalin* OR memantin* OR amantadin* OR ketamin* OR esketamin* OR dextromethorphan* OR agmatin* OR minocyclin* OR acetylcystein* OR NAC OR glycin* OR cycloserin* OR sarcosin* OR carnosin* OR riluzol* OR modafinil*
3	random* OR trial OR placebo
4	#1 AND #2 AND #3

© 2025 Coelho DRA et al. *JAMA Network Open.*

eTable 4. Full Search Strategy for Web of Science

1	TI=(OCRD OR OCD OR obsess* OR compulsi* OR "body dysmorphic disorder" OR BDD OR "image disorder" OR "image dysfunction" OR trichotillomania OR "hair-pulling" OR trichobezoar OR excoriation OR "skin-picking" OR hoarding OR "pathological collecting" OR hoarder)
2	AB=(OCRD OR OCD OR obsess* OR compulsi* OR "body dysmorphic disorder" OR BDD OR "image disorder" OR "image dysfunction" OR trichotillomania OR "hair-pulling" OR trichobezoar OR excoriation OR "skin-picking" OR hoarding OR "pathological collecting" OR hoarder)
3	AK=(OCRD OR OCD OR obsess* OR compulsi* OR "body dysmorphic disorder" OR BDD OR "image disorder" OR "image dysfunction" OR trichotillomania OR "hair-pulling" OR trichobezoar OR excoriation OR "skin-picking" OR hoarding OR "pathological collecting" OR hoarder)
4	#1 OR #2 OR #3
5	TI=("excitatory amino acid agents" OR glutamat* OR NMDA OR AMPA OR lamotrigin* OR topiramat* OR gabapentin* OR pregabalin* OR memantin* OR amantadin* OR ketamin* OR esketamin* OR dextromethorphan* OR agmatin* OR minocyclin* OR acetylcystein* OR NAC OR glycin* OR cycloserin* OR sarcosin* OR carnosin* OR riluzol* OR modafinil*)
6	AB=("excitatory amino acid agents" OR glutamat* OR NMDA OR AMPA OR lamotrigin* OR topiramat* OR gabapentin* OR pregabalin* OR memantin* OR amantadin* OR ketamin* OR esketamin* OR dextromethorphan* OR agmatin* OR minocyclin* OR acetylcystein* OR NAC OR glycin* OR cycloserin* OR sarcosin* OR carnosin* OR riluzol* OR modafinil*)
7	AK=("excitatory amino acid agents" OR glutamat* OR NMDA OR AMPA OR lamotrigin* OR topiramat* OR gabapentin* OR pregabalin* OR memantin* OR amantadin* OR ketamin* OR esketamin* OR dextromethorphan* OR agmatin* OR minocyclin* OR acetylcystein* OR NAC OR glycin* OR cycloserin* OR sarcosin* OR carnosin* OR riluzol* OR modafinil*)
8	#5 OR #6 OR #7
9	TI=(random* OR trial OR placebo)
10	AB=(random* OR trial OR placebo)
11	AK=(random* OR trial OR placebo)
12	#9 OR #10 OR #11
13	#4 AND #8 AND #12

© 2025 Coelho DRA et al. *JAMA Network Open.*

eTable 5. Full Search Strategy for Cochrane Central Register of Controlled Trials

1	OCRD OR OCD OR obsess* OR compulsi* OR "body dysmorphic" OR BDD OR "image disorder" OR "image dysfunction" OR trichotillomania OR "hair-pulling" OR trichobezoar OR excoriation OR "skin picking" OR hoarding OR "pathological collecting" OR hoarder
2	"excitatory amino acid agents" OR glutamat* OR NMDA OR AMPA OR lamotrigin* OR topiramat* OR gabapentin* OR pregabalin* OR memantin* OR amantadin* OR ketamin* OR esketamin* OR dextromethorphan* OR agmatin* OR minocyclin* OR acetylcystein* OR NAC OR glycin* OR cycloserin* OR sarcosin* OR carnosin* OR riluzol* OR modafinil*
3	#1 AND #2

© 2025 Coelho DRA et al. *JAMA Network Open.*

eTable 6. List of Excluded Studies

Author (Year)	Title	Reason
Aguiar et al. 2021	Adjunctive troriluzole-a novel glutamate modulator-in patients with obsessive compulsive disorder: impact of baseline disease severity on treatment outcomes	Abstract
Andersson et al. 2015	D-Cycloserine vs Placebo as Adjunct to Cognitive Behavioral Therapy for Obsessive-Compulsive Disorder and Interaction With Antidepressants: A Randomized Clinical Trial.	Due to intervention
Bakhshaie et al. 2020	Temporal precedence of the change in obsessive-compulsive symptoms and change in depressive symptoms during exposure and response prevention for pediatric obsessive-compulsive disorders.	Due to intervention
Berlin et al. 2008	Double-blind, placebo-controlled trial of topiramate augmentation in the treatment of obsessive-compulsive disorder	Abstract
Berlin et al. 2011	Double-blind, placebo-controlled trial of topiramate augmentation in treatment-resistant obsessive-compulsive disorder.	Due to outcome
Bloch et al. 2012	N-acetylcysteine in the treatment of pediatric trichotillomania: a randomized, double-blind, placebo-controlled trial	Abstract
Bloch et al. 2016	N-Acetylcysteine in the Treatment of Pediatric Tourette Syndrome: Randomized, Double-Blind, Placebo-Controlled Add-On Trial.	Due to population
Chamberlain et al. 2010	Effects of acute modafinil on cognition in trichotillomania.	Due to study design
Chasson et al. 2010	Need for speed: evaluating slopes of OCD recovery in behavior therapy enhanced with d-cycloserine.	Due to intervention
Costa et al. 2015	Serotonin reuptake inhibitor augmentation with n-acetylcysteine in treatment resistant OCD: a double-blind randomized controlled trial	Abstract
deLeeuw et al. 2017	D-cycloserine addition to exposure sessions in the treatment of patients with obsessive-compulsive disorder.	Due to intervention
Farnia et al. 2018	Efficacy and tolerability of adjunctive gabapentin and memantine in obsessive compulsive disorder: Double-blind, randomized, placebo-controlled trial.	Due to study design
Farrell et al. 2014	A randomised controlled trial to evaluate the effects of D-Cycloserine in combination with intensive exposure therapy, versus pill placebo in combination with intensive exposure therapy in the treatment of pediatric Obsessive Compulsive Disorder to impro	Due to intervention
Farrell et al. 2022	Efficacy of D-cycloserine augmented brief intensive cognitive-behavioural therapy for paediatric obsessive-compulsive disorder: A randomised clinical trial.	Due to intervention
Geller et al. 2011	2/2 D-Cycloserine Augmentation of CBT for Pediatric OCD	Abstract
Geller et al. 2019	Fear extinction learning as a predictor of response to cognitive behavioral therapy for pediatric obsessive compulsive disorder.	Due to intervention

© 2025 Coelho DRA et al. *JAMA Network Open.*

Ghanizadeh et al. 2013	N-acetylcysteine versus placebo for treating nail biting, a double blind randomized placebo controlled clinical trial	Due to outcome
Grant et al. 2010	Neurocognitive predictors of lamotrigine treatment for pathologic skin picking: a doubleblind, placebo-controlled trial	Abstract
Grant et al. 2015	A double-blind, placebo-controlled trial of N-acetyl cysteine in the treatment of skin picking disorder	Abstract
Grant et al. 2017	Riluzole Serum Concentration in Pediatric Patients Treated for Obsessive-Compulsive Disorder.	Due to outcome
Grant et al. 2023	Double-Blind Placebo-Controlled Study of Memantine in Trichotillomania and Skin-Picking Disorder.	Due to outcome
Guzick et al. 2021	Irritability in Children and Adolescents With OCD.	Due to intervention
Haghighi et al. 2013	In a double-blind, randomized and placebo-controlled trial, adjuvant memantine improved symptoms in inpatients suffering from refractory obsessive-compulsive disorders (OCD)	Abstract
Henin et al. 2017	Long-term efficacy of cognitive-behavioral therapy for pediatric OCD with and without d-cycloserine augmentation	Abstract
Joseph et al. 2011	A placebo-controlled trial of riluzole for treatment of childhood-onset obsessive compulsive disorder	Abstract
Koran et al. 2005	Double-blind treatment with oral morphine in treatment-resistant obsessive-compulsive disorder.	Due to intervention
Kushner et al. 2007	D-cycloserine augmented exposure therapy for obsessive-compulsive disorder.	Due to intervention
Kvale et al. 2020	Effect of D-Cycloserine on the Effect of Concentrated Exposure and Response Prevention in Difficult-to-Treat Obsessive-Compulsive Disorder: A Randomized Clinical Trial.	Due to intervention
Loew et al. 2006	Topiramate treatment for women with borderline personality disorder: a double-blind, placebo-controlled study.	Due to population
Mataix-Cols et al. 2014	Cognitive-behavioural therapy with post-session D-cycloserine augmentation for paediatric obsessive-compulsive disorder: pilot randomised controlled trial.	Due to intervention
McGuire et al. 2019	Defining Treatment Outcomes in Pediatric Obsessive-Compulsive Disorder Using a Self-Report Scale.	Due to intervention
Nematizadeh et al. 2023	L-theanine combination therapy with fluvoxamine in moderate-to-severe obsessive-compulsive disorder: A placebo-controlled, double-blind, randomized trial.	Due to intervention
NicolideMattos et al. 2020	A 12-Week Randomized, Double-Blind, Placebo-Controlled Clinical Trial of Topiramate for the Treatment of Compulsive Buying Disorder.	Due to population
Niemeyer et al. 2022	Memantine as treatment for compulsivity in child and adolescent psychiatry: Descriptive findings from an incompleted randomized, double-blind, placebo-controlled trial.	Due to population
O'Neill et al. 2017	Glutamate in Pediatric Obsessive-Compulsive Disorder and Response to Cognitive-Behavioral Therapy: Randomized Clinical Trial.	Due to intervention

© 2025 Coelho DRA et al. *JAMA Network Open.*

Olatunji et al. 2015	Effects of Homework Compliance on Cognitive-Behavioral Therapy with D-Cycloserine Augmentation for Children with Obsessive Compulsive Disorder.	Due to intervention
Olson et al. 2022	Two Randomized, Double-Blind, Placebo-Controlled Trials of Adjunctive Troriluzole, a Novel Glutamate Modulating Agent, in Obsessive Compulsive Disorder	Abstract
Park et al. 2014	Does d-Cycloserine Augmentation of CBT Improve Therapeutic Homework Compliance for Pediatric Obsessive-Compulsive Disorder?	Due to intervention
Pittenger et al. 2007	A Double-blind Study of Riluzole Augmentation in Serotonin Reuptake Inhibitor-refractory Obsessive-compulsive Disorder and Depression	Protocol
Rodriguez et al. 2012	A randomized controlled crossover trial of ketamine in obsessive-compulsive disorder	Abstract
Rodriguez et al. 2013	Randomized controlled crossover trial of ketamine in obsessive-compulsive disorder: proof-of-concept.	Due to study design
Rodriguez et al. 2017	Challenges testing intranasal ketamine in obsessive-compulsive disorder (OCD)	Due to study design
Rodriguez et al. 2022	Efficacy of Ketamine in Unmedicated Adults With Obsessive-Compulsive Disorder: a Randomized Controlled Trial	Abstract
Rubio et al. 2006	The effects of topiramate adjunctive treatment added to antidepressants in patients with resistant obsessive-compulsive disorder.	Due to study design
Rück et al. 2012	Internet-based Cognitive Behavior Therapy in Combination With D-Cycloserine for Obsessive Compulsive Disorder: a Double Blinded Randomized Controlled Trial	Due to intervention
Rynn et al. 2018	Minocycline Augmentation of Serotonin Reuptake Inhibitors in Pediatric OCD: Randomized Control Trial to Test Feasibility, Acceptability, Efficacy, and Striatal Glutamate Effects	Abstract
Sahraian et al. 2014	Topiramate as an adjuvant treatment for obsessive compulsive symptoms in patients with bipolar disorder: a randomized double blind placebo controlled clinical trial.	Due to population
Sahraian et al. 2017	Memantine as an Adjuvant Treatment for Obsessive Compulsive Symptoms in Manic Phase of Bipolar Disorder: A Randomized, Double-Blind, Placebo-Controlled Clinical Trial.	Due to population
Sarris et al. 2016	Participant characteristics as modifiers of response to N-acetyl cysteine (NAC) in obsessive-compulsive disorder	Due to outcome
Schumer et al. 2015	Long-Term Outcome in Pediatric Trichotillomania.	Due to study design
Storch et al. 2007	D-cycloserine does not enhance exposure-response prevention therapy in obsessive-compulsive disorder.	Due to intervention
Storch et al. 2010	D-cycloserine augmentation of cognitive-behavioral therapy in pediatric obsessive-compulsive disorder: a preliminary study	Abstract
Storch et al. 2016	Efficacy of Augmentation of Cognitive Behavior Therapy With Weight-Adjusted d-Cycloserine vs Placebo in Pediatric Obsessive-Compulsive Disorder: A Randomized Clinical Trial.	Due to intervention

© 2025 Coelho DRA et al. *JAMA Network Open.*

Storch et al. 2016	Augmentation of cognitive behavioral therapy with d-cycloserine in pediatric obsessive-compulsive disorder: a randomized controlled trial	Abstract
Tundo et al. 2009	Effectiveness of the addition of cognitive-behavioral therapy for medication non-responder patients with obsessive compulsive disorder. A 12-month naturalistic study	Report not in English
VanRoessel et al. 2022	Cognitive Control Predicts Alleviation of OCD Symptoms by Ketamine	Abstract
vanRoessel et al. 2023	538. Cognitive Control Predicts Alleviation of OCD Symptoms by Ketamine	Abstract
Weingarden et al. 2019	D-Cycloserine-Augmented Behavior Therapy for Body Dysmorphic Disorder: A Preliminary Efficacy Trial	Due to intervention
Welter et al. 2021	Deep brain stimulation of the subthalamic, accumbens, or caudate nuclei for patients with severe obsessive-compulsive disorder: A randomized crossover controlled study	Due to intervention
Wilhelm et al. 2003	A Medication Trial Combined With Behavior Therapy for Individuals With Obsessive-compulsive Disorder	Protocol
Wilhelm et al. 2008	Augmentation of behavior therapy with D-cycloserine for obsessive-compulsive disorder.	Due to intervention
Wilhelm et al. 2018	D-Cycloserine augmentation of cognitive behavior therapy for pediatric OCD: Predictors and moderators of outcome.	Due to intervention

© 2025 Coelho DRA et al. *JAMA Network Open.*

eFigure 1. Cochrane Risk of Bias for Randomized Controlled Trials by Domains

Risk of bias domains

Study	D1	D2	D3	D4	D5	Overall
Afshar et al. 2012	+	+	+	+	+	+
Afshar et al. 2014	+	+	-	+	+	-
Askari et al. 2022	+	+	X	+	+	X
Arabzadeh et al. 2017	+	+	+	+	+	+
Bloch et al. 2013	+	+	-	+	+	-
Bruno et al. 2012	+	+	+	+	+	+
Costa et al. 2017	+	+	-	+	+	-
Emmazadehfard et al. 2016	+	+	+	+	+	+
Esalatmanesh et al. 2016	+	+	+	+	+	+
Ghaleiha et al. 2013	+	+	+	+	+	+
Ghanizadeh et al. 2017	+	+	+	+	+	+
Grant et al. 2009	+	+	+	+	+	+
Grant et al. 2010	+	+	+	+	+	+
Grant et al. 2014	+	+	+	+	+	+
Grant et al. 2016	+	+	X	+	+	X
Greenberg et al. 2009	+	+	-	+	+	-
Haghighi et al. 2013	+	+	X	+	+	X
Khalkhali et al. 2016	+	+	+	+	+	+
Li et al. 2020	+	+	+	+	+	+
Modaressi et al. 2018	+	+	+	+	+	+
Mowla et al. 2010	+	+	-	+	+	-
Mowla et al. 2019	+	+	-	+	+	-
Naderi et al. 2019	+	+	+	+	+	+
Paydary et al. 2016	+	+	+	+	+	+
Pittenger et al. 2015	+	+	+	+	+	+
Sarris et al. 2015	+	+	+	+	+	+
Sarris et al. 2022	+	-	-	+	+	-

Domains:
D1: Bias arising from the randomization process.
D2: Bias due to deviations from intended intervention.
D3: Bias due to missing outcome data.
D4: Bias in measurement of the outcome.
D5: Bias in selection of the reported result.

Judgement
X High
- Some concerns
+ Low

© 2025 Coelho DRA et al. *JAMA Network Open.*

eFigure 2. Cochrane Risk of Bias for Randomized Controlled Trials Overall

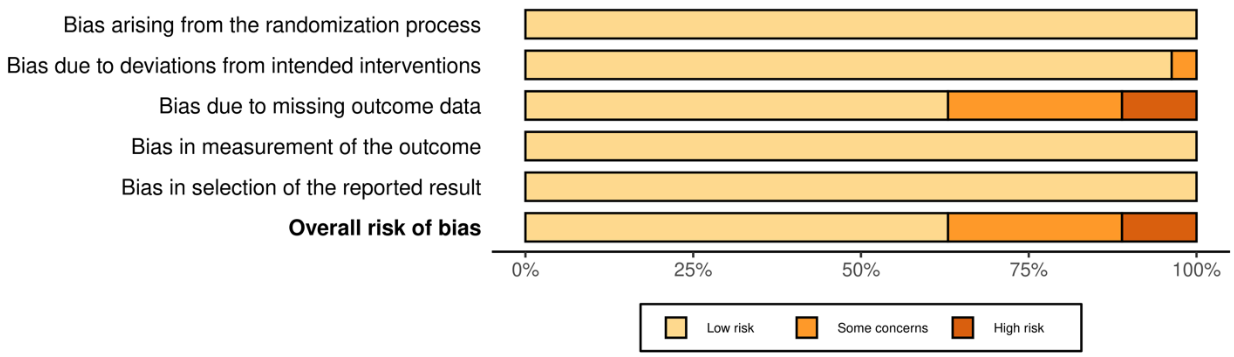

© 2025 Coelho DRA et al. *JAMA Network Open.*

eTable 7. Summary of GRADE

Certainty assessment							№ of patients		Effect		Certainty	Importance
№ of studies	Study design	Risk of bias	Inconsistency	Indirectness	Imprecision	Other considerations	[Intervention]	[Control]	Relative (95% CI)	Absolute (95% CI)		
**Glutamatergic medications for obsessive-compulsive and related disorders (OCRDs)**												
27	randomized trials	not serious	very serious[a]	not serious	not serious	publication bias strongly suspected[b] strong association	688	681	-	SMD **0.8 SD lower** (1.13 lower to 0.47 higher)	⨁⨁◯◯ Low	IMPORTANT
**Glutamatergic medications for obsessive-compulsive disorder (OCD)**												
23	randomized trials	not serious	very serious[a]	not serious	not serious	strong association	592	590	-	MD 4.17 **SD lower** (5.82 lower to 2.52 lower)	⨁⨁⨁◯ Moderate	IMPORTANT

CI: confidence interval; **MD:** mean difference; **SMD:** standardized mean difference

Risk of bias: None of the outcomes had a majority of the studies categorized as having some concerns or high risk of bias.
Inconsistency: We assessed the consistency using I^2 estimates and through a visual inspection of the point estimates.
Indirectness: None of the outcomes were informed by indirect comparisons.
Imprecision: We assessed imprecision by analyzing whether the confidence intervals included values less than the minimal important difference, indicating uncertainty about the clinical significance of the treatment effect.

Explanations
a. Considerable heterogeneity was observed, and the point estimates demonstrated notable inconsistency across the studies.
b. Publication bias was strongly suspected based on the asymmetry of the funnel plot and the statistical significance of Egger's test.

© 2025 Coelho DRA et al. *JAMA Network Open.*

eFigure 3. Meta-Analysis Obsessive-Compulsive and Related Disorders (OCRDs): Funnel Plot

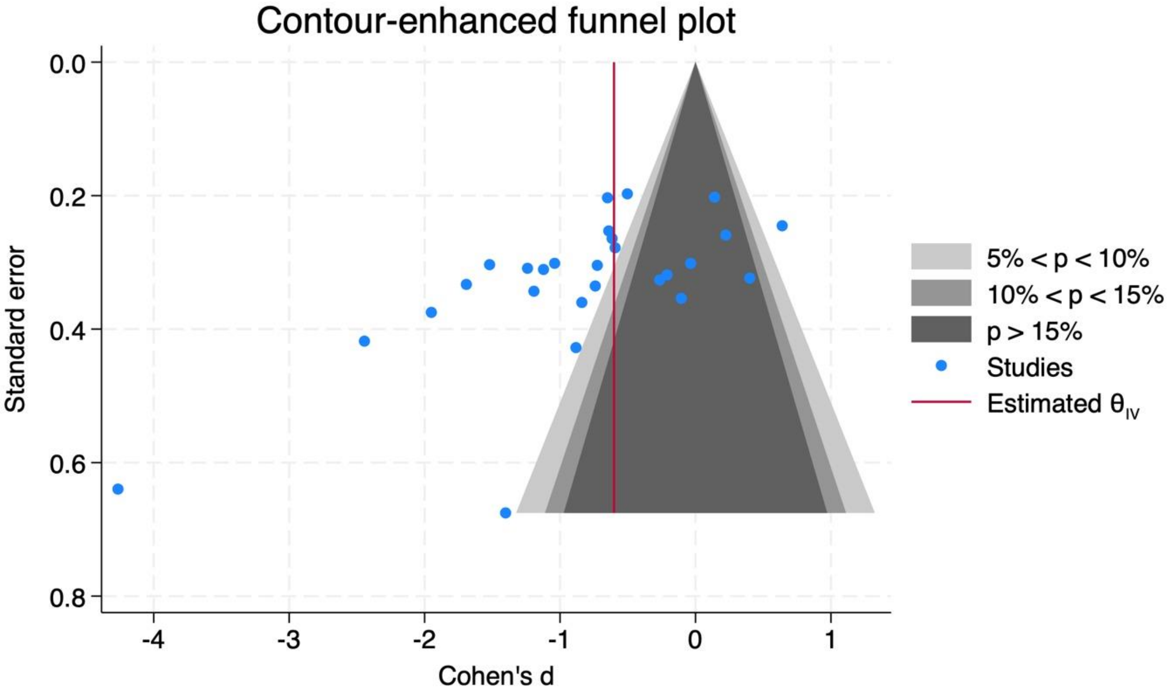

© 2025 Coelho DRA et al. *JAMA Network Open.*

eFigure 4. Subgroup Analysis Based on Type of OCRD

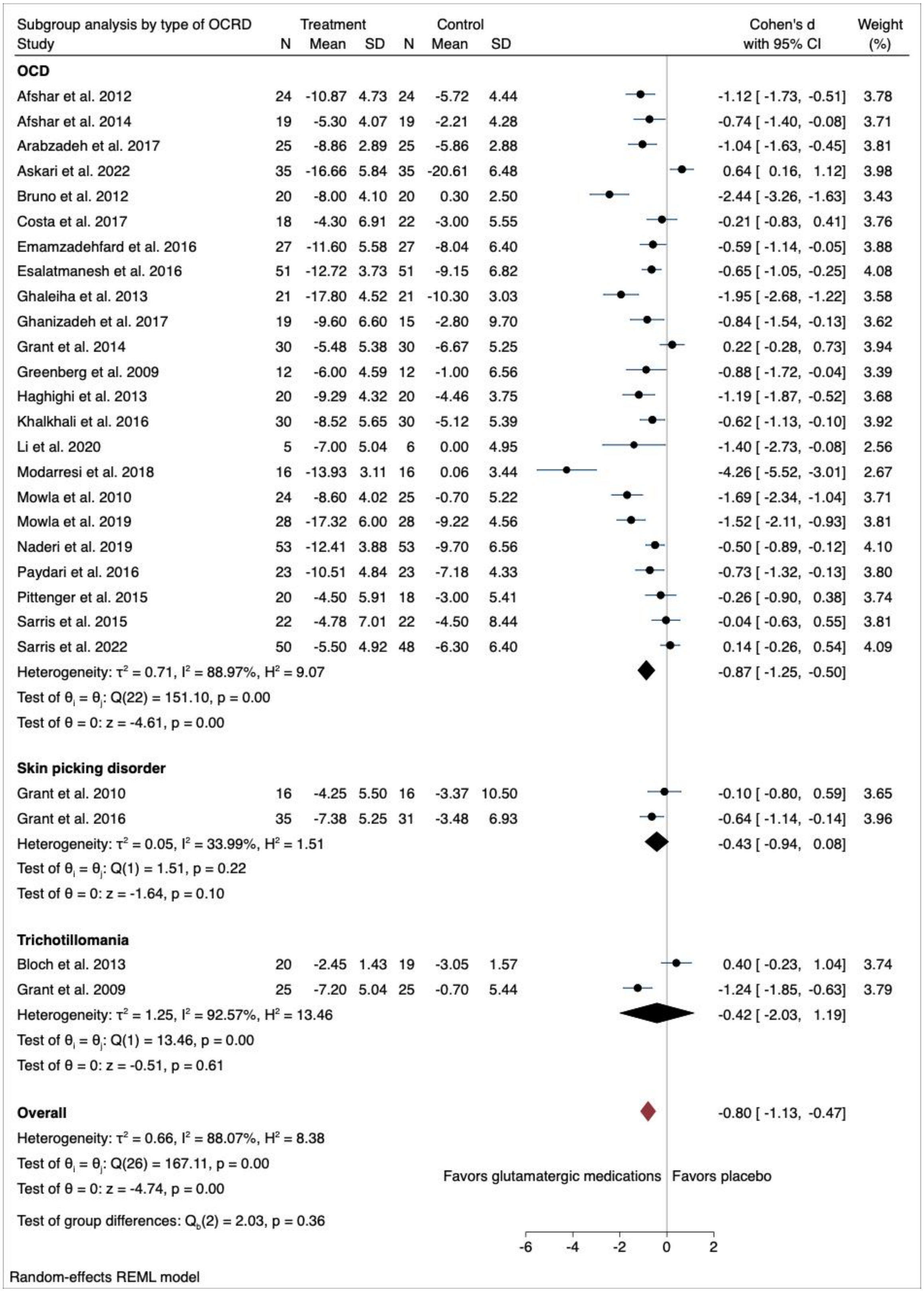

© 2025 Coelho DRA et al. *JAMA Network Open.*

eFigure 5. Subgroup Analysis Based on Population

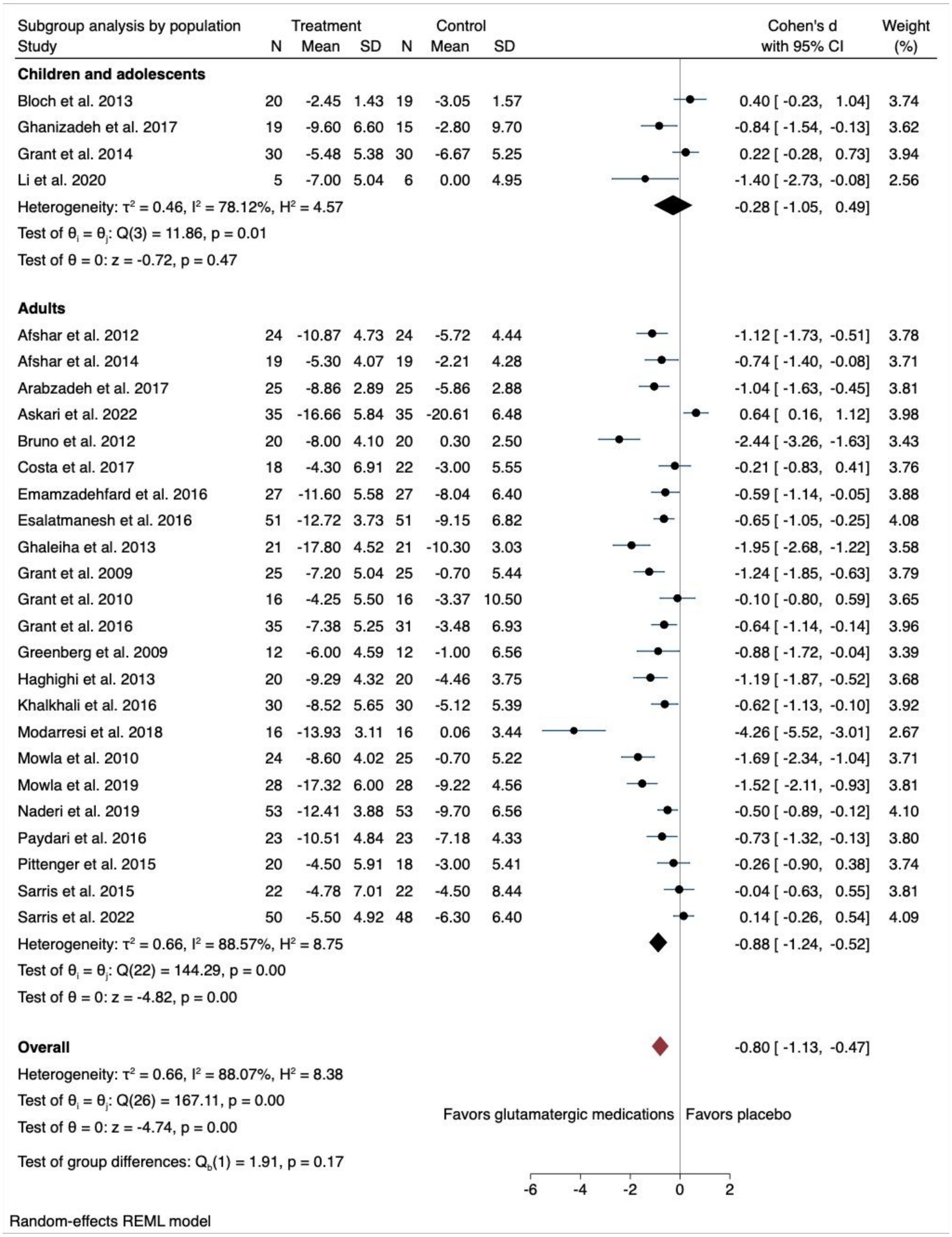

© 2025 Coelho DRA et al. *JAMA Network Open.*

eFigure 6. Subgroup Analysis Based on Refractoriness of OCRD

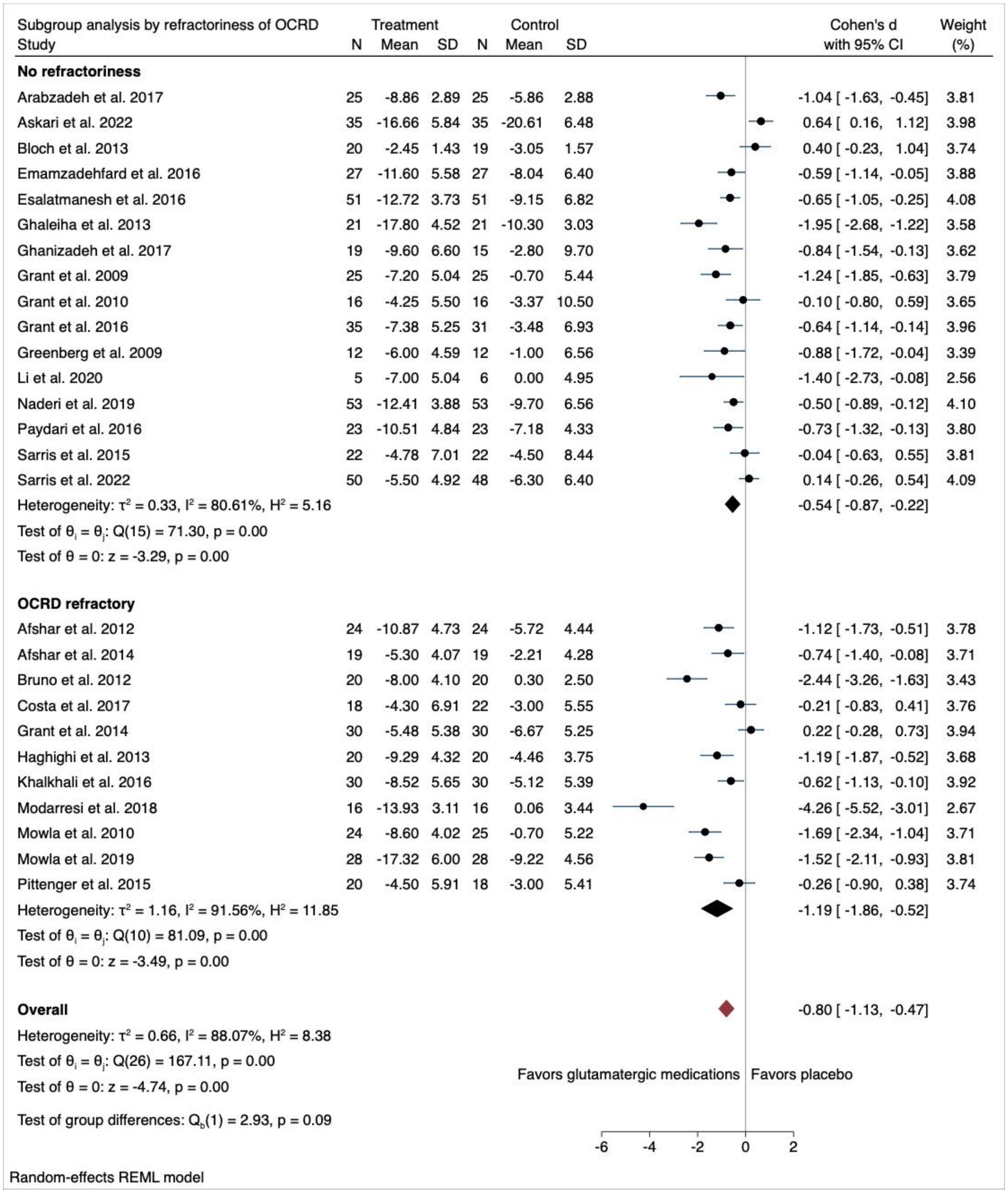

© 2025 Coelho DRA et al. *JAMA Network Open.*

eFigure 7. Subgroup Analysis Based on Augmentation Strategy

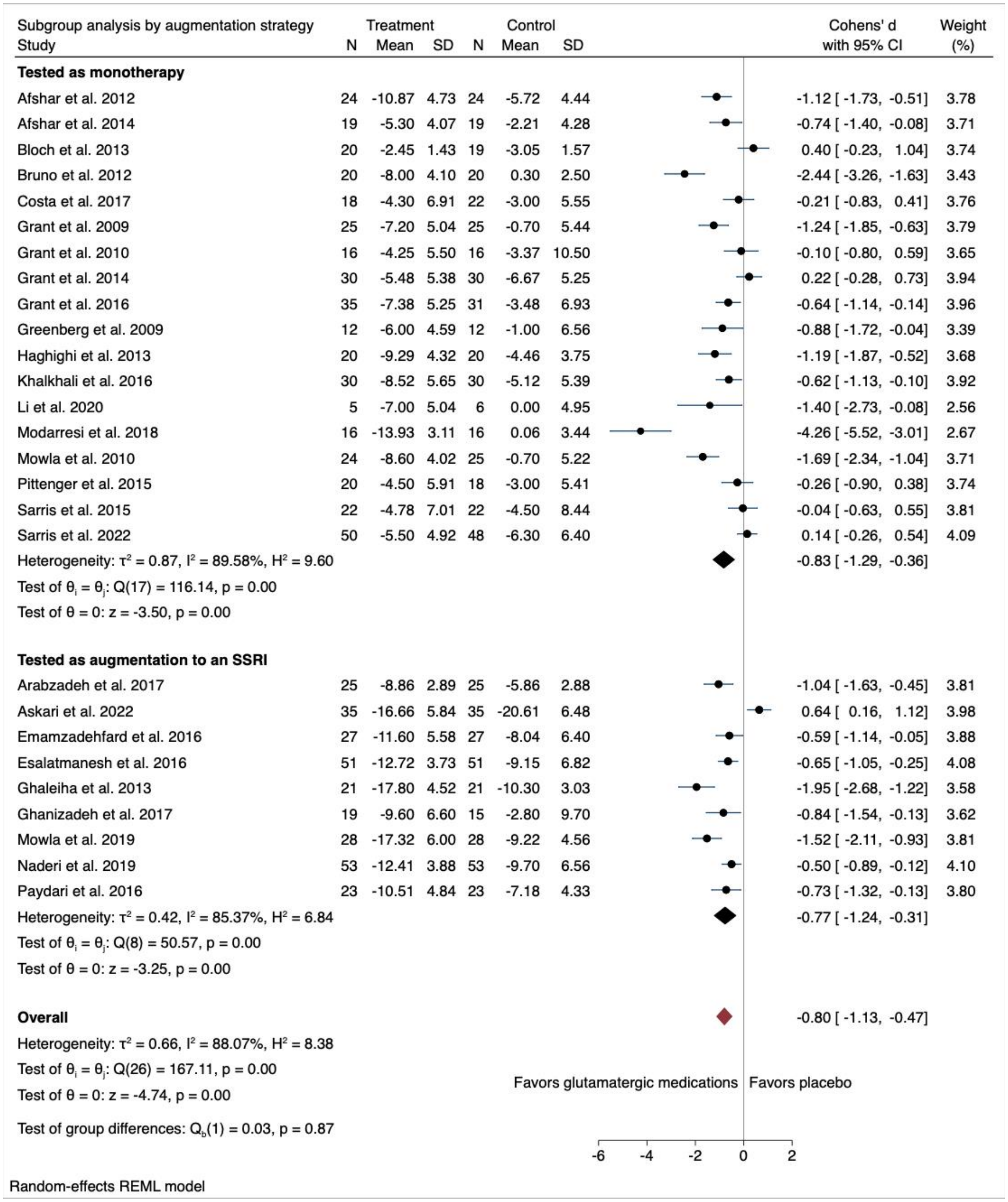

© 2025 Coelho DRA et al. *JAMA Network Open.*

eFigure 8. Subgroup Analysis Based on Risk of Bias

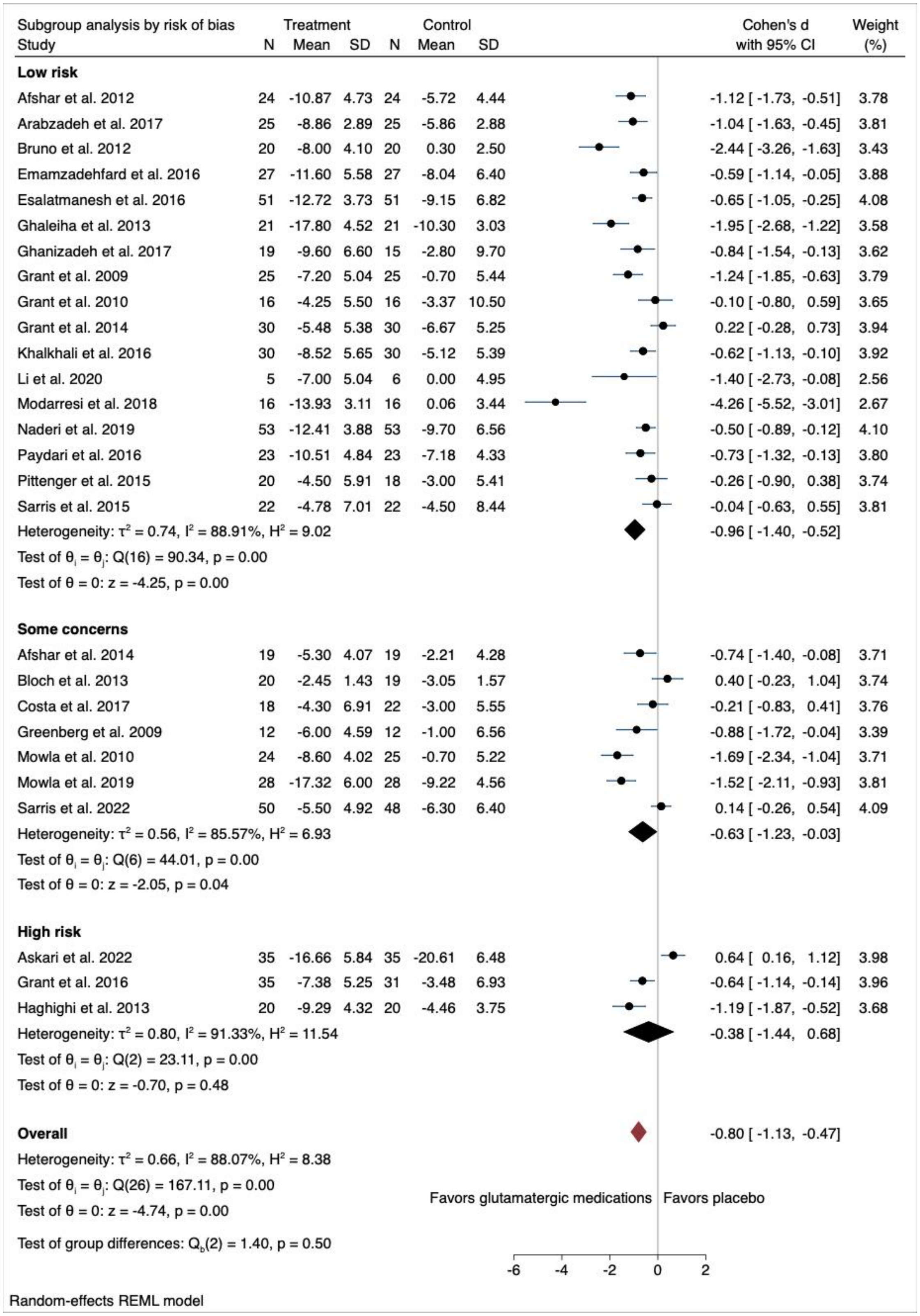

© 2025 Coelho DRA et al. *JAMA Network Open.*

eFigure 9. Subgroup Analysis Based on Type of Glutamatergic Medication

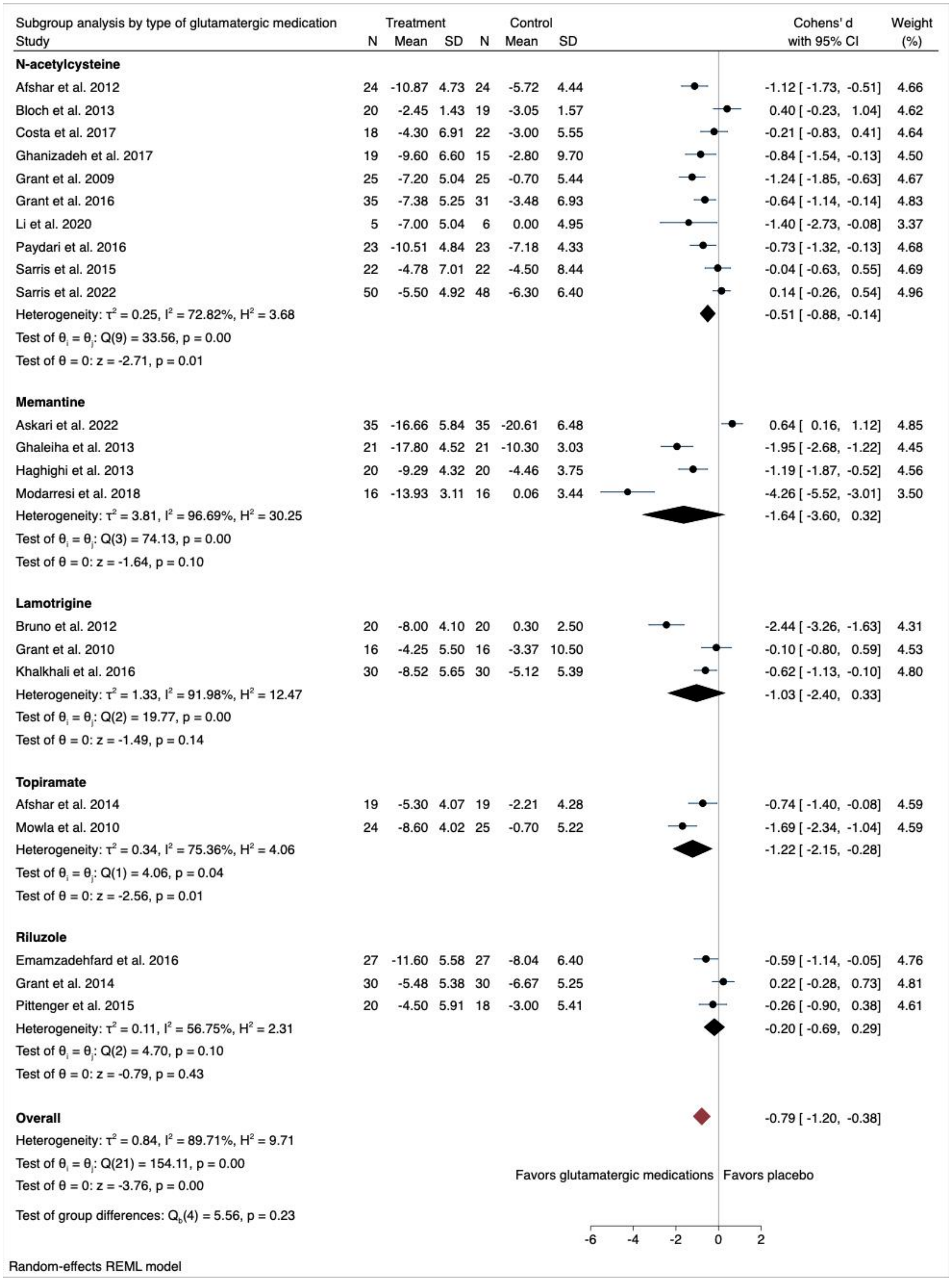

© 2025 Coelho DRA et al. *JAMA Network Open.*

eFigure 10. Sensitivity Analysis Using a Leave-One-Out Analysis

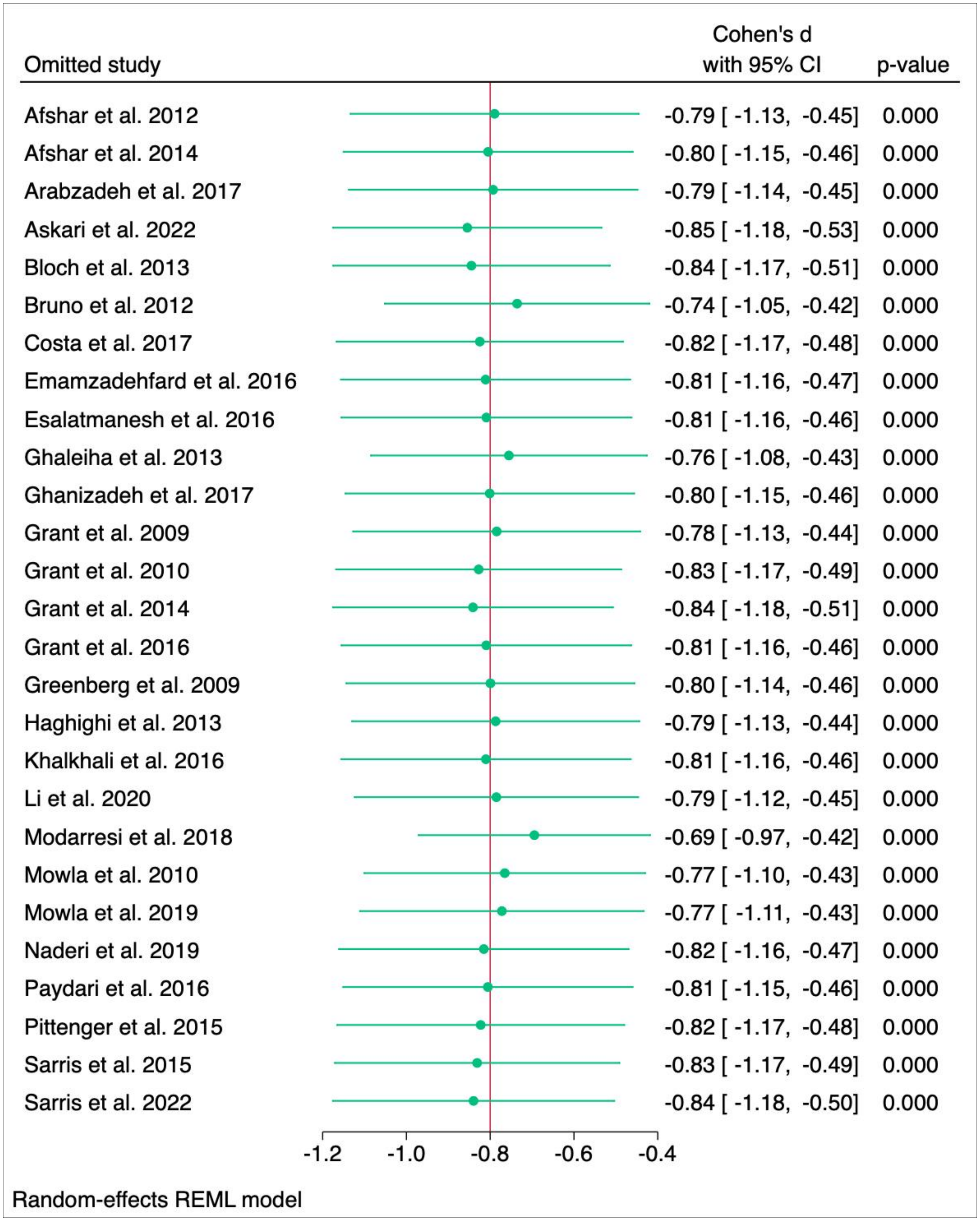

© 2025 Coelho DRA et al. *JAMA Network Open.*

eTable 8. Univariate Meta-Regression by Mean Age, Mean Years Living With OCRD, or Weeks of Treatment

Variable	Coefficient	95% CI	p-value
Mean age (years)	-0.019	-0.063, 0.025	0.405
Mean years living with OCRD	0.077	-0.043, 0.196	0.210
Weeks of treatment	0.078	-0.053, 0.209	0.244

© 2025 Coelho DRA et al. *JAMA Network Open.*

eFigure 11. Meta-Analysis Obsessive-Compulsive Disorder (OCD): Funnel Plot

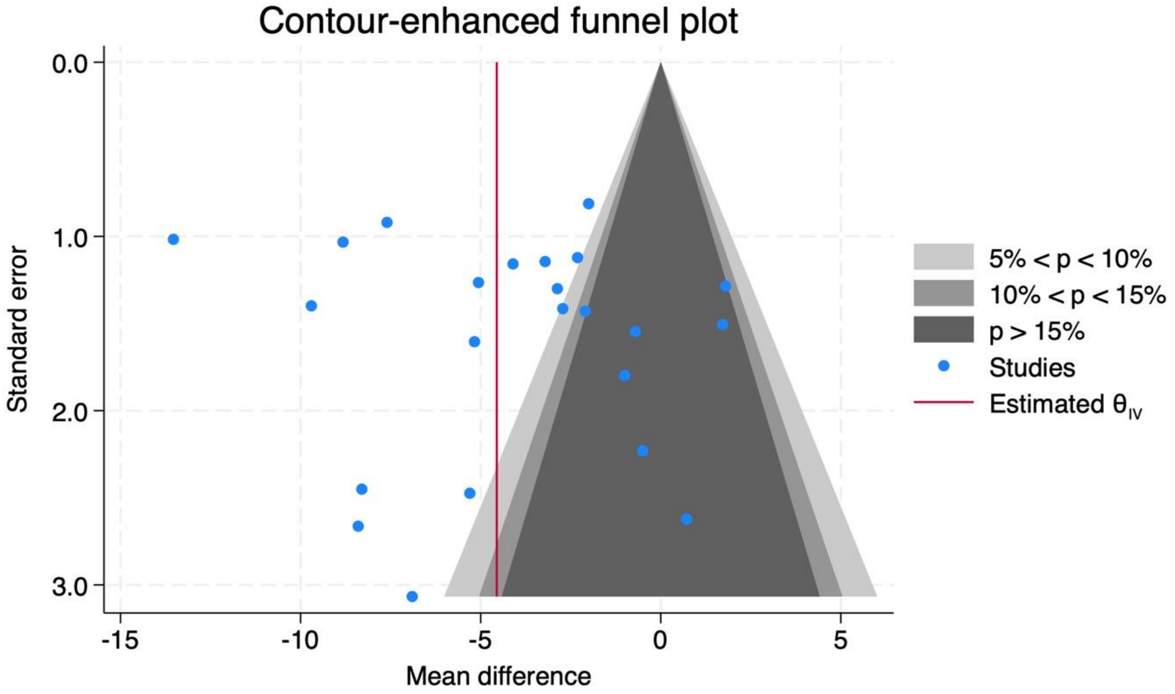

© 2025 Coelho DRA et al. *JAMA Network Open.*

eFigure 12. Subgroup Analysis Based on Population

Subgroup analysis by population / Study	Treatment N	Treatment Mean	Treatment SD	Control N	Control Mean	Control SD	Mean difference with 95% CI	Weight (%)
**Children and adolescents**								
Ghanizadeh et al. 2017	19	11.30	5.70	15	19.70	9.70	-8.40 [-13.62, -3.18]	3.43
Grant et al. 2014	30	21.76	6.28	30	22.46	5.68	-0.70 [-3.73, 2.33]	4.44
Li et al. 2020	5	14.40	5.55	6	21.30	4.64	-6.90 [-12.91, -0.89]	3.09
Heterogeneity: $\tau^2 = 14.11$, $I^2 = 71.53\%$, $H^2 = 3.51$							-4.87 [-9.93, 0.19]	
Test of $\theta_i = \theta_j$: Q(2) = 7.87, p = 0.02								
Test of $\theta = 0$: z = -1.89, p = 0.06								
**Adults**								
Afshar et al. 2012	24	16.83	4.38	24	21.89	4.38	-5.06 [-7.54, -2.58]	4.67
Afshar et al. 2014	19	21.45	3.44	19	24.32	4.50	-2.87 [-5.42, -0.32]	4.64
Arabzadeh et al. 2017	25	16.23	3.20	25	18.23	2.50	-2.00 [-3.59, -0.41]	4.98
Askari et al. 2022	35	11.22	6.26	35	9.50	6.34	1.72 [-1.23, 4.67]	4.47
Bruno et al. 2012	20	17.65	9.70	20	25.95	5.10	-8.30 [-13.10, -3.50]	3.62
Costa et al. 2017	18	21.30	8.10	22	21.80	6.00	-0.50 [-4.87, 3.87]	3.82
Emamzadehfard et al. 2016	27	17.12	4.30	27	19.84	5.96	-2.72 [-5.49, 0.05]	4.55
Esalatmanesh et al. 2016	51	17.21	3.57	51	20.42	7.35	-3.21 [-5.45, -0.97]	4.76
Ghaleiha et al. 2013	21	10.80	2.74	21	18.40	3.20	-7.60 [-9.40, -5.80]	4.91
Greenberg et al. 2009	12	18.40	4.80	12	23.70	7.10	-5.30 [-10.15, -0.45]	3.60
Haghighi et al. 2013	20	19.57	3.76	20	23.67	3.56	-4.10 [-6.37, -1.83]	4.75
Khalkhali et al. 2016	30	18.31	6.60	30	23.48	5.80	-5.17 [-8.31, -2.03]	4.39
Modarresi et al. 2018	16	20.00	2.56	16	33.53	3.16	-13.53 [-15.52, -11.54]	4.85
Mowla et al. 2010	24	17.90	4.30	25	27.60	5.40	-9.70 [-12.44, -6.96]	4.56
Mowla et al. 2019	28	8.81	3.47	28	17.63	4.22	-8.82 [-10.84, -6.80]	4.84
Naderi et al. 2019	53	17.62	4.01	53	19.93	7.11	-2.31 [-4.51, -0.11]	4.78
Paydari et al. 2016	23	16.53	5.36	23	18.63	4.27	-2.10 [-4.90, 0.70]	4.54
Pittenger et al. 2015	20	25.10	6.19	18	26.10	4.69	-1.00 [-4.52, 2.52]	4.22
Sarris et al. 2015	22	21.77	8.04	22	21.05	9.31	0.72 [-4.42, 5.86]	3.47
Sarris et al. 2022	50	17.60	5.65	48	15.80	7.02	1.80 [-0.72, 4.32]	4.65
Heterogeneity: $\tau^2 = 14.30$, $I^2 = 89.43\%$, $H^2 = 9.46$							-4.08 [-5.87, -2.29]	
Test of $\theta_i = \theta_j$: Q(19) = 197.03, p = 0.00								
Test of $\theta = 0$: z = -4.47, p = 0.00								
**Overall**							-4.17 [-5.82, -2.52]	
Heterogeneity: $\tau^2 = 13.62$, $I^2 = 88.08\%$, $H^2 = 8.39$								
Test of $\theta_i = \theta_j$: Q(22) = 205.97, p = 0.00								
Test of $\theta = 0$: z = -4.95, p = 0.00								
Test of group differences: $Q_b(1) = 0.08$, p = 0.77								

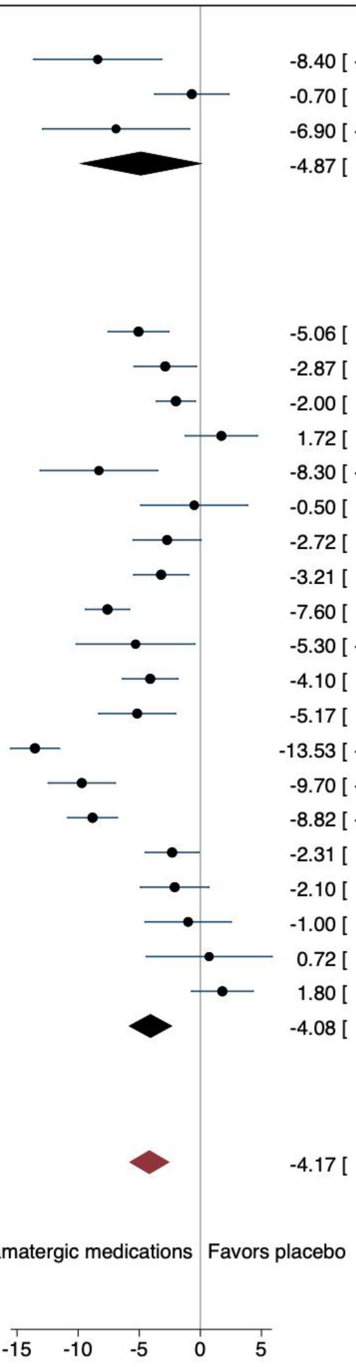

Random-effects REML model

© 2025 Coelho DRA et al. *JAMA Network Open.*

eFigure 13. Subgroup Analysis Based on Refractoriness of OCD

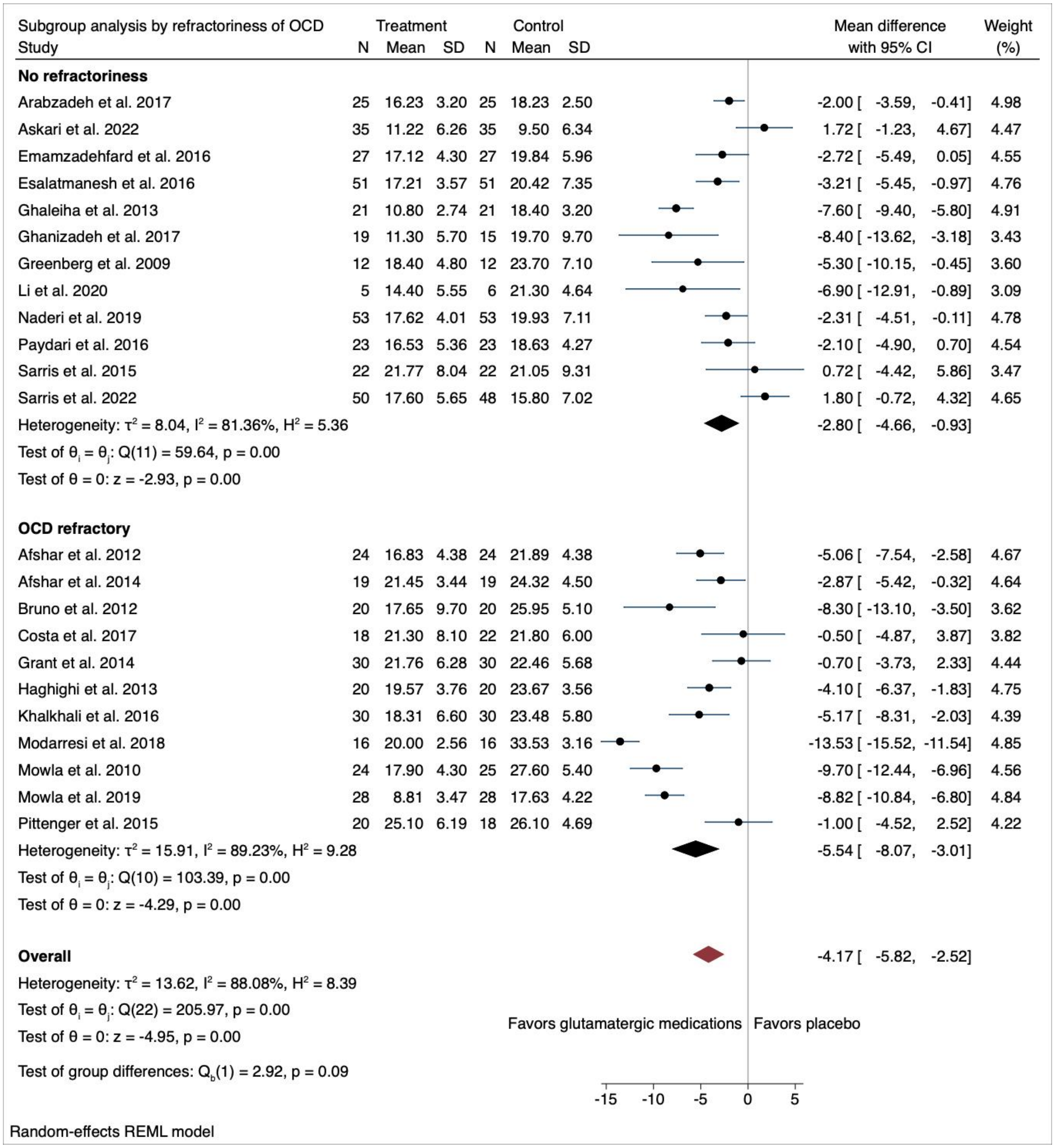

© 2025 Coelho DRA et al. *JAMA Network Open.*

eFigure 14. Subgroup Analysis Based on Augmentation Strategy

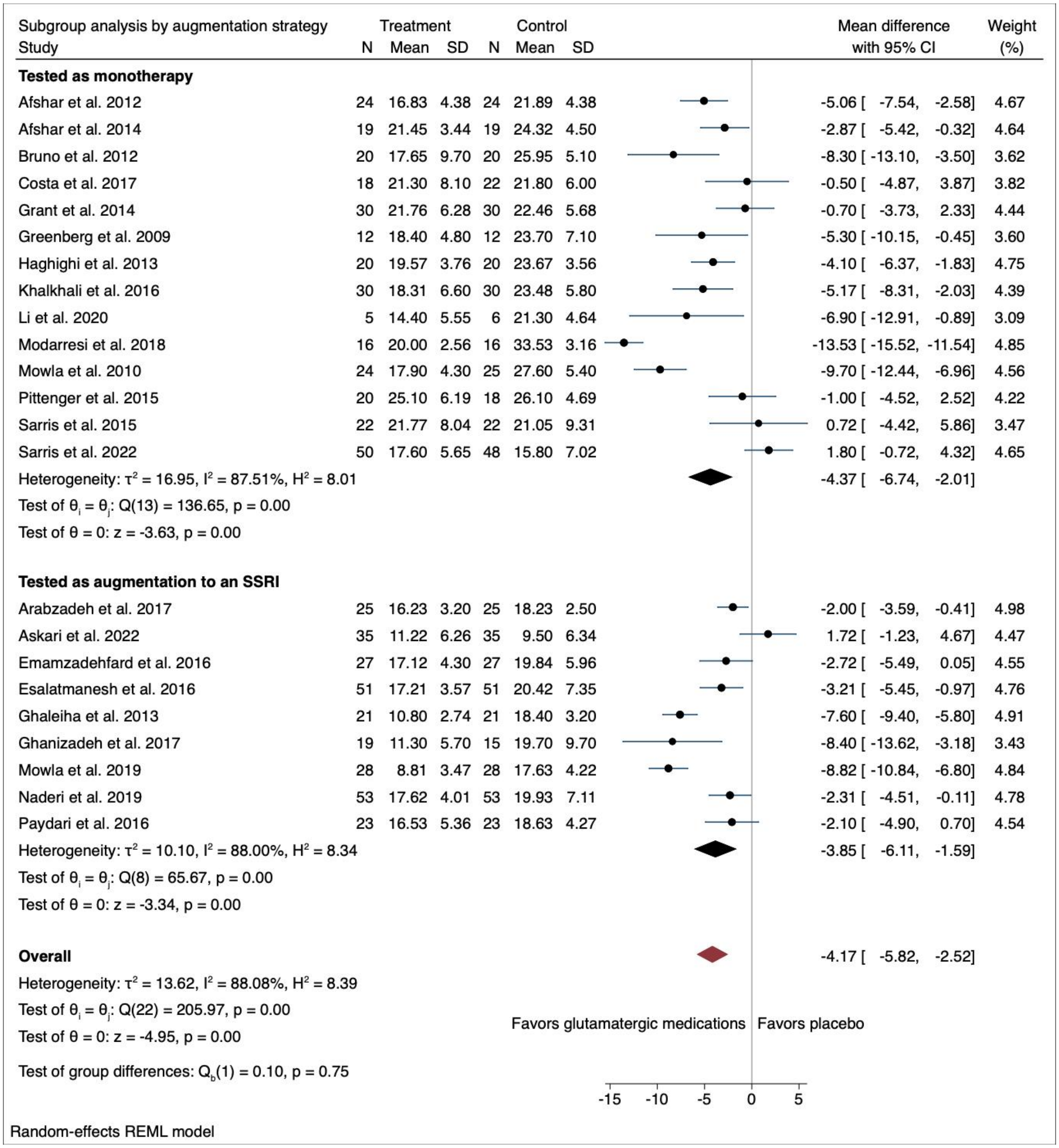

© 2025 Coelho DRA et al. *JAMA Network Open.*

eFigure 15. Subgroup Analysis Based on Risk of Bias

Subgroup analysis by risk of bias	Treatment			Control			Mean difference	Weight
Study	N	Mean	SD	N	Mean	SD	with 95% CI	(%)
**Low risk**								
Afshar et al. 2012	24	16.83	4.38	24	21.89	4.38	-5.06 [-7.54, -2.58]	4.67
Arabzadeh et al. 2017	25	16.23	3.20	25	18.23	2.50	-2.00 [-3.59, -0.41]	4.98
Bruno et al. 2012	20	17.65	9.70	20	25.95	5.10	-8.30 [-13.10, -3.50]	3.62
Emamzadehfard et al. 2016	27	17.12	4.30	27	19.84	5.96	-2.72 [-5.49, 0.05]	4.55
Esalatmanesh et al. 2016	51	17.21	3.57	51	20.42	7.35	-3.21 [-5.45, -0.97]	4.76
Ghaleiha et al. 2013	21	10.80	2.74	21	18.40	3.20	-7.60 [-9.40, -5.80]	4.91
Ghanizadeh et al. 2017	19	11.30	5.70	15	19.70	9.70	-8.40 [-13.62, -3.18]	3.43
Grant et al. 2014	30	21.76	6.28	30	22.46	5.68	-0.70 [-3.73, 2.33]	4.44
Khalkhali et al. 2016	30	18.31	6.60	30	23.48	5.80	-5.17 [-8.31, -2.03]	4.39
Li et al. 2020	5	14.40	5.55	6	21.30	4.64	-6.90 [-12.91, -0.89]	3.09
Modarresi et al. 2018	16	20.00	2.56	16	33.53	3.16	-13.53 [-15.52, -11.54]	4.85
Naderi et al. 2019	53	17.62	4.01	53	19.93	7.11	-2.31 [-4.51, -0.11]	4.78
Paydari et al. 2016	23	16.53	5.36	23	18.63	4.27	-2.10 [-4.90, 0.70]	4.54
Pittenger et al. 2015	20	25.10	6.19	18	26.10	4.69	-1.00 [-4.52, 2.52]	4.22
Sarris et al. 2015	22	21.77	8.04	22	21.05	9.31	0.72 [-4.42, 5.86]	3.47
Heterogeneity: $\tau^2 = 12.26$, $I^2 = 87.04\%$, $H^2 = 7.72$							-4.52 [-6.49, -2.55]	
Test of $\theta_i = \theta_j$: Q(14) = 127.85, p = 0.00								
Test of $\theta = 0$: z = -4.51, p = 0.00								
**Some concerns**								
Afshar et al. 2014	19	21.45	3.44	19	24.32	4.50	-2.87 [-5.42, -0.32]	4.64
Costa et al. 2017	18	21.30	8.10	22	21.80	6.00	-0.50 [-4.87, 3.87]	3.82
Greenberg et al. 2009	12	18.40	4.80	12	23.70	7.10	-5.30 [-10.15, -0.45]	3.60
Mowla et al. 2010	24	17.90	4.30	25	27.60	5.40	-9.70 [-12.44, -6.96]	4.56
Mowla et al. 2019	28	8.81	3.47	28	17.63	4.22	-8.82 [-10.84, -6.80]	4.84
Sarris et al. 2022	50	17.60	5.65	48	15.80	7.02	1.80 [-0.72, 4.32]	4.65
Heterogeneity: $\tau^2 = 19.56$, $I^2 = 90.27\%$, $H^2 = 10.28$							-4.30 [-8.08, -0.52]	
Test of $\theta_i = \theta_j$: Q(5) = 59.75, p = 0.00								
Test of $\theta = 0$: z = -2.23, p = 0.03								
**High risk**								
Askari et al. 2022	35	11.22	6.26	35	9.50	6.34	1.72 [-1.23, 4.67]	4.47
Haghighi et al. 2013	20	19.57	3.76	20	23.67	3.56	-4.10 [-6.37, -1.83]	4.75
Heterogeneity: $\tau^2 = 15.13$, $I^2 = 89.35\%$, $H^2 = 9.39$							-1.27 [-6.97, 4.43]	
Test of $\theta_i = \theta_j$: Q(1) = 9.39, p = 0.00								
Test of $\theta = 0$: z = -0.44, p = 0.66								
**Overall**							-4.17 [-5.82, -2.52]	
Heterogeneity: $\tau^2 = 13.62$, $I^2 = 88.08\%$, $H^2 = 8.39$								
Test of $\theta_i = \theta_j$: Q(22) = 205.97, p = 0.00								
Test of $\theta = 0$: z = -4.95, p = 0.00								
Test of group differences: $Q_b(2) = 1.12$, p = 0.57								

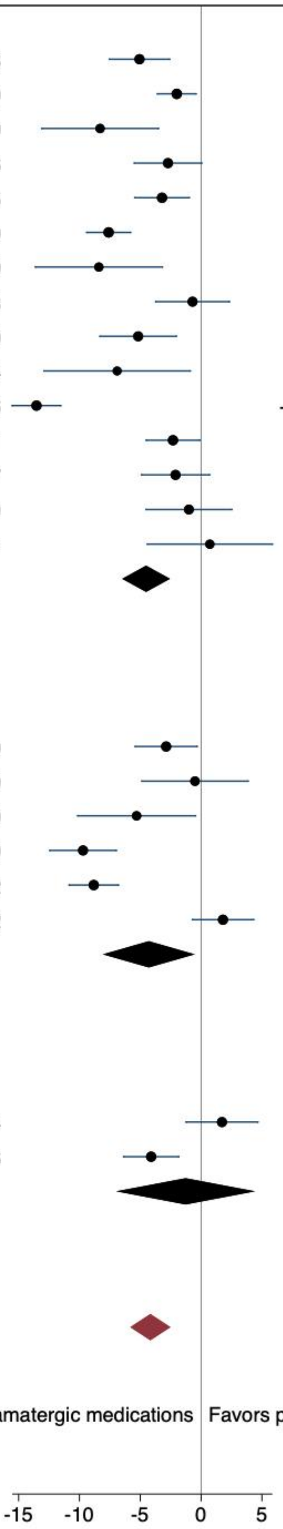

Random-effects REML model

© 2025 Coelho DRA et al. *JAMA Network Open.*

eFigure 16. Subgroup Analysis Based on Type of Glutamatergic Medication

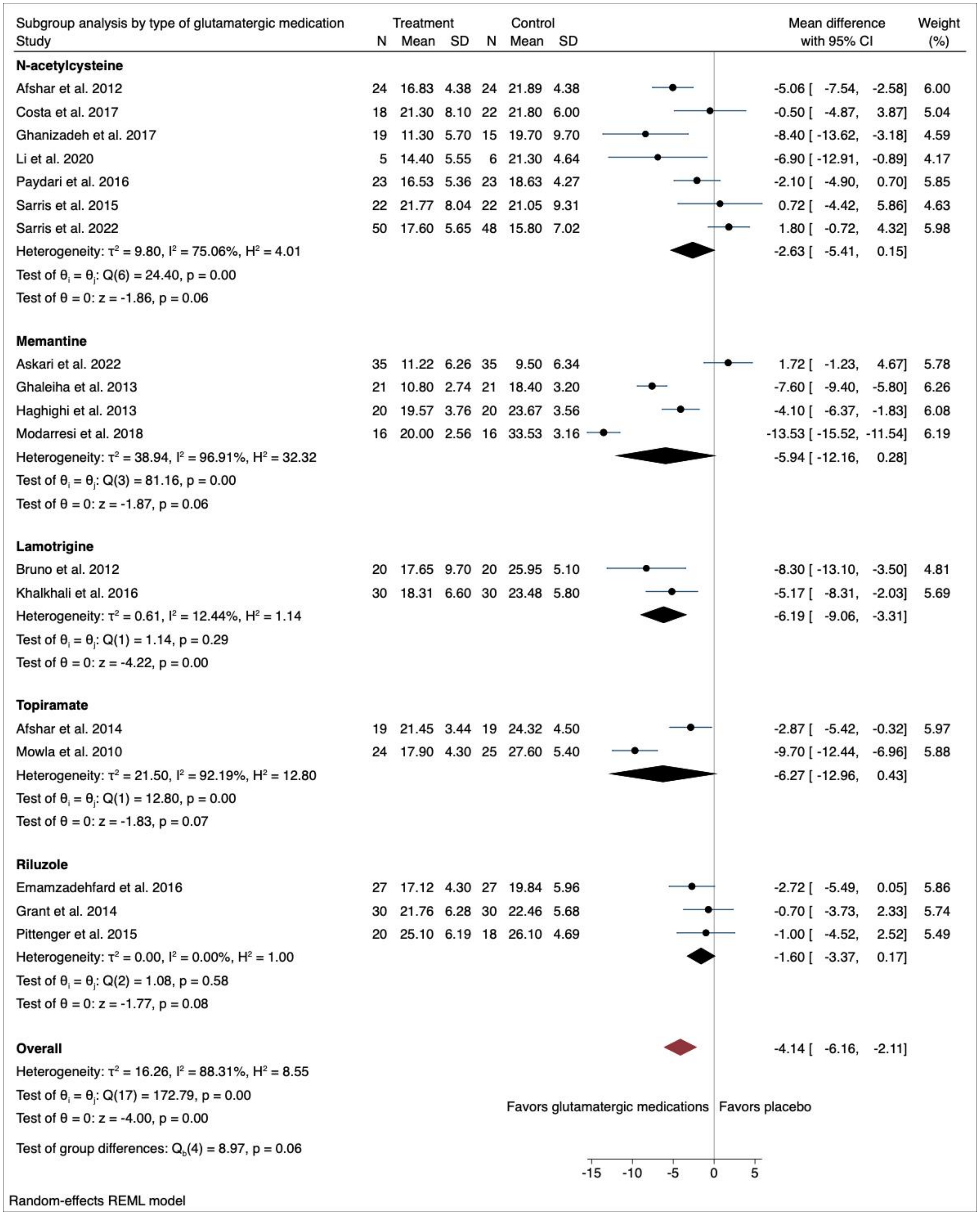

© 2025 Coelho DRA et al. *JAMA Network Open.*

eFigure 17. Sensitivity Analysis Using a Leave-One-Out Analysis

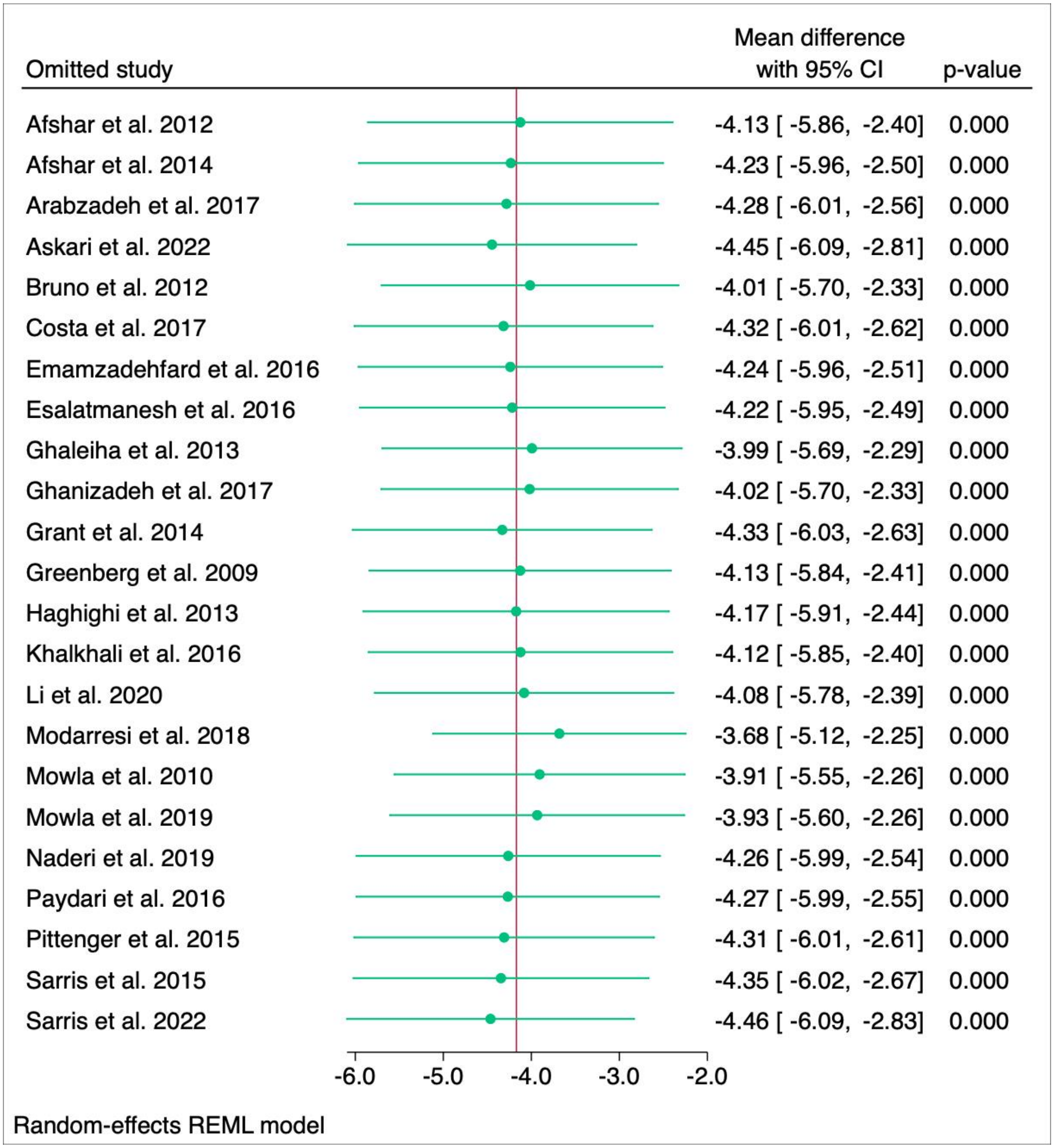

© 2025 Coelho DRA et al. *JAMA Network Open.*

eTable 9. Univariate Meta-Regression by Mean Age, Mean Years Living With OCD, or Weeks of Treatment

Variable	Coefficient	95% CI	p-value
Mean age (years)	0.078	-0.160, 0.315	0.522
Mean years living with OCD	0.359	-0.119, 0.837	0.141
Weeks of treatment	0.502	-0.107, 1.111	0.106

© 2025 Coelho DRA et al. *JAMA Network Open.*
