# Supplementary material for: Glutamatergic Medications for Obsessive-Compulsive and Related Disorders: A Systematic Review and Meta-Analysis
Source: JAMA Netw Open. 2025 Jan 2;8(1):e2452963. doi: 10.1001/jamanetworkopen.2024.52963 (PMC11696454; doi:10.1001/jamanetworkopen.2024.52963)
Supplement: Supplement 2. — Data Sharing Statement [file jamanetwopen-e2452963-s002.pdf]

Data Sharing Statement

Coelho. Glutamatergic Medications for Obsessive-Compulsive and Related Disorders. *JAMA Netw Open*. Published January 02, 2025. doi:10.1001/jamanetworkopen.2024.52963

Data
Data available: No

Additional Information
Explanation for why data not available: Data available: No Explanation for why data not available: All data is publicly available, as this is a systematic review of existing studies. A list of all included studies is available in the references.
